# Effects of dietary chromium supplementation on dry matter intake and milk production and composition in lactating dairy cows: A meta-analysis

**DOI:** 10.3389/fvets.2023.1076777

**Published:** 2023-03-16

**Authors:** Muhammad I. Malik, Didier Raboisson, Xin Zhang, Xuezhao Sun

**Affiliations:** ^1^The Innovation Centre of Ruminant Precision Nutrition and Smart Farming, Jilin Agricultural Science and Technology University, Jilin, China; ^2^Department of Animal Nutrition, The University of Veterinary and Animal Sciences, Lahore, Pakistan; ^3^Université de Toulouse, École Nationale Vétérinaire de Toulouse (ENVT), Toulouse, France; ^4^College of Biological and Pharmaceutical Engineering, Jilin Agricultural Science and Technology University, Jilin, China; ^5^Jilin Inter-Regional Cooperation Centre for the Scientific and Technological Innovation of Ruminant Precision Nutrition and Smart and Ecological Farming, Jilin, China; ^6^AgResearch Limited, Grasslands Research Centre, Palmerston North, New Zealand

**Keywords:** chromium supplementation, dairy cows, lactation, dry matter intake, milk production, milk composition, meta-analysis

## Abstract

**Introduction:**

Chromium (Cr) is an essential mineral that has been demonstrated to enhance milk production in dairy cows. This study aims to evaluate the effects of dietary Cr supplementation on dry matter intake (DMI), milk production and composition using a meta-analysis based on existing literature.

**Methods:**

A random effects meta-analysis was performed to investigate the effects of dietary Cr supplementation on DMI, milk production and composition. The heterogeneity was assessed using the *I*^2^ statistic and Q test, while Egger's test was used to evaluate publication bias.

**Results:**

The meta-analysis discovered that Cr-supplemented cows had a significantly higher DMI compared to those not supplemented, with an increase of 0.72 kg/day [95% confidence interval (CI), 0.46–0.97]. The regression model indicated that DMI significantly increased by 0.9 g/kg of body weight (BW) and by 80.5 g for an increase of 1 mg of Cr supplement. The supplementation phase was associated with an increase in DMI, with an increase of 0.4582 kg/day for BFP (before parturition) and 0.853 kg/day for AFP (after parturition). The methionine and yeast forms of Cr increased DMI by 0.714 and 1.137 kg/day, respectively. The DMI was increased by 2.137 and 0.620 kg/day for multiparous (MP)+ primiparous (PP) cows and MP cows, respectively. Milk production was also increased by Cr supplementation, with an increase of 1.20 kg/day (95% CI, 0.65–1.76). The regression model indicated that milk production increased by 2.3 g/day for an increase of 1 kg of BW and by 122.4 g/day for an increase of 1 mg of Cr supplement. Milk production also increased with the duration of the experiment and days in milk. The amino acid and methionine forms of Cr complexes increased milk production by 1.645 and 1.448 kg/day, respectively. Milk production increased by 1.087 and 1.920 kg/day for MP and PP cows, respectively. Milk composition was not significantly affected by Cr supplementation. Egger's test for publication biases was not significant for all responses of interest.

**Discussion:**

The meta-analysis showed that Cr supplementation improves DMI and milk production in dairy cows. The results suggest that the supplementation phase, form of Cr, and parity should be considered when supplementing dairy cows with Cr. The results have important implications for the dairy industry and can contribute to the development of more effective feeding strategies for dairy cows.

## 1. Introduction

With the increase in global demand for dairy products, ruminant nutritionists are looking for metabolic modifiers to improve the production performance of dairy cattle. To attain high performance in milking cows, ensuring an optimal balance of all nutrients including microminerals is an important segment in the feed additive industry.

Chromium (Cr) is a micromineral. Elemental Cr was discovered in 1798 (1). Since then, Cr has been found with several beneficial effects on the organism. It was identified as an essential mineral in rats (2) and its essentiality was further established in several animal studies (3–5). In the late 1990s, Cr also started to be studied intensively as an essential mineral in livestock animals (cattle, sheep, horses, pigs, and poultry) (6).

In dairy cows, dietary Cr supplementation is found to improve dry matter intake (DMI), milk production, and milk composition of dairy cows in the early, mid, or late stages of lactation (7). This essential mineral can modify glucose and lipid metabolisms (7) and improve antioxidant and immune functions (8). Chromium requirements increase under physiological stress (9–11). Feed ingredients commonly available for dairy cows have a low concentration of Cr. To fulfill the requirements of Cr, Cr-containing additives are often added to the diet of cows.

Although Cr is widely regarded as an essential mineral and often supplemented in the diet, effects of Cr supplementation on DMI, milk production, and milk composition are inconsistent in dairy cows. Al-Saiady et al. (12) and Wu et al. (13) reported that Cr improved milk yield. On the contrary, Leiva et al. (14) reported no effects on milk production. The parity of the cows influences the effect of Cr supplementation. Primiparous cows had a positive response in terms of milk production, but multiparous cows did not show any effects (12). The dose of Cr supplementation also had varying results on DMI and milk production (7). Smith et al. (15) reported that DMI and milk production of multiparous dairy cows in early lactation increased when Cr was added to the diet. Kafilzadeh and Targhibi (16) investigated the effect of Cr supplementation on performance of multiparous dairy cows in early lactation and reported that milk production was not affected.

Due to these inconsistencies in previously published literature, meta-analysis was performed to evaluate the effects of dietary Cr supplementation on DMI, milk production and milk composition of dairy cows.

## 2. Materials and methods

### 2.1. Literature search and selection criteria

The comprehensive data search was carried out with PUBMED (https://www.ncbi.nlm.nih.gov/pubmed/), Agricola (https://agricola.nal.usda.gov/), CABI (https://www.cabi.org/publishing-products/animal-science-database/).

**Figure 1:**
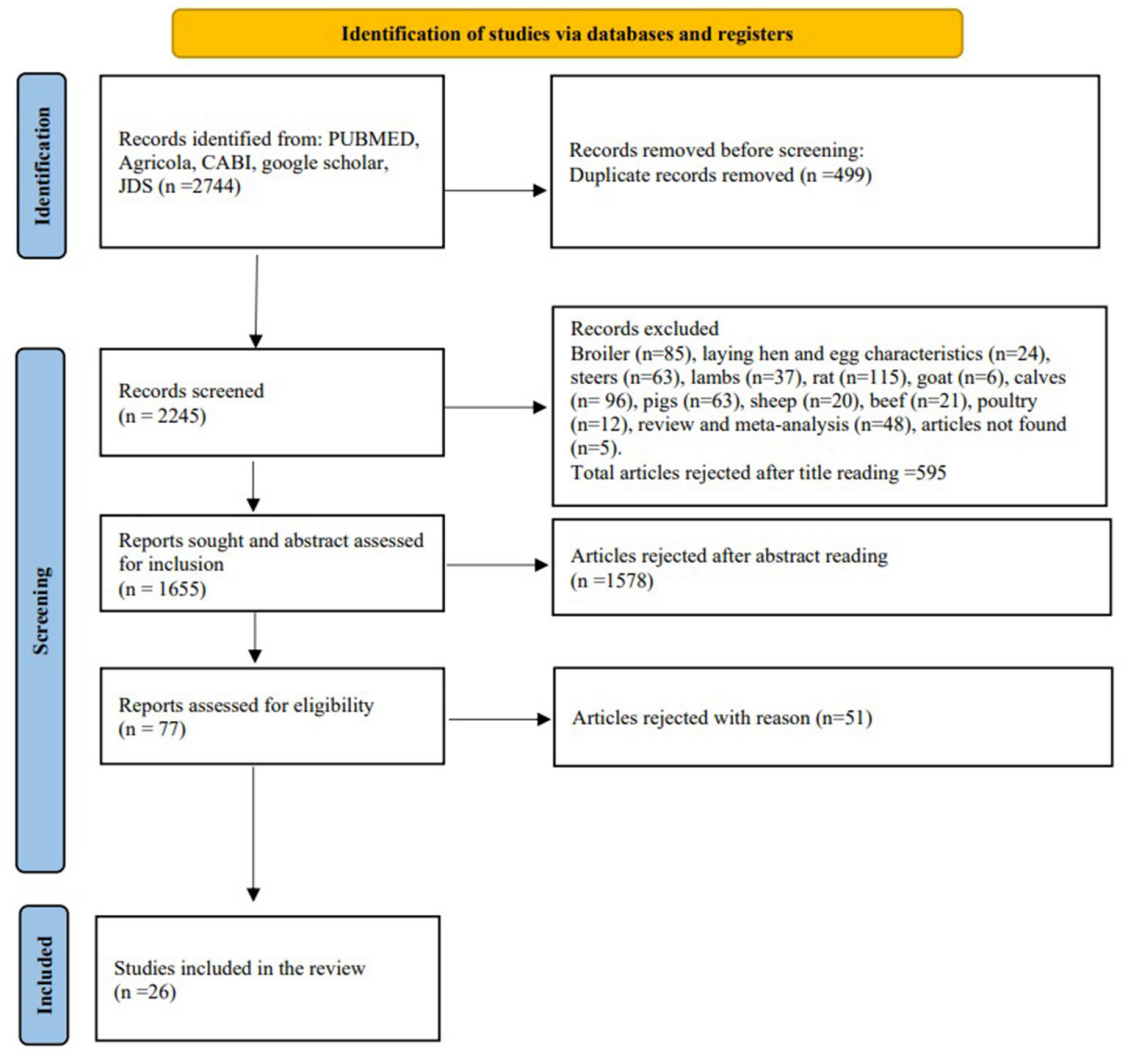
The flow diagram of search strategies, data screening, exclusion, and inclusion of the studies in meta-analysis.

Scopus (https://www.scopus.com) and Google Scholar (https://scholar.google.com/) databases. The keywords used to create datasets in each search engine included “chromium and cattle”, “chromium and cow”, “chromium and cows”, “chromium and dairy cow”, “chromium and dairy cattle”, “chromium supplementation”, “chromium supplementation and cattle”, “chromium supplementation and cow”, “chromium supplementation and cows”, “chromium supplementation and dairy cow”, “chromium supplementation and dairy cows”, and “chromium supplementation and dairy cattle”. For Google scholar, we searched each keyword for up to 10 pages. Additionally, we searched the Journal of Dairy Science (https://www.journalofdairyscience.org/action/doSearch?text1=chromium&field1=AbstractTitleKeywordFilterField) with only a single keyword “chromium” by applying filters to article title, abstract and keywords. The data of each parameter [DMI, milk production, milk fat percent, milk protein percent, milk lactose percent, and solids-not-fat (SNF) percent] were extracted only if the response of interest was evaluated during Cr supplementation. The data were excluded if Cr was supplemented for less than a week. The data were also excluded if the recording was done after supplementation of Cr had stopped because in some studies milk production and milk composition parameters were also evaluated after cessation of Cr supplementation to evaluate the carryover effects of treatments. Additionally, data on body weight (BW), experimental duration/duration of Cr supplementation, parity of the cows, days in milk (DIM), stage of parturition [before (BFP) or after (AFP) parturition], Cr complexes (amino acids, yeast, picolinate, or propionate) were extracted from studies. The studies without variance (SE or SD), mean or data given in figures without means provided were also rejected. The overview of the studies included or excluded in the meta-analysis is provided in a flow chart (Figure 1). There are totally 26 studies (8, 12–36) included for the meta-analysis.

### 2.2. Statistical analysis

The statistical analysis was conducted using the mean difference (MD) as the outcome of the measure. A random-effect model was fitted to the data. A random-effect model allowed the true effect to vary from study to study and include between-study variability (true heterogeneity) as well as sampling error (37). The outcome variables were DMI, milk production, milk fat content, milk protein content, milk lactose content, and SNF content. The forest plots were developed to visualize the summary effects of the random effects meta-analytical model for each outcome variables using the R package dmetar (version 0.0.9000) (38). The amount of heterogeneity (τ^2^) was estimated using the restricted maximum-likelihood estimator. Tau-squared is a measure of heterogeneity in meta-analysis. It is the variance of the true effects across studies and represents the amount of variability in the results that is not explained by random error. A larger tau-squared value indicates greater heterogeneity among the studies, suggesting that the results are more dispersed and may not be generalizable to a larger population. In meta-analysis, tau-squared is often used in random-effects models to weigh the contribution of each study to the overall estimate of effect size (39).

In addition to the estimate of τ^2^, the *Q*-test for heterogeneity (40) and the *I*^2^ statistic (41) were reported. The *I*_2_ value was defined as *I*^2^ = (Q – df/Q) × 100, where Q is the χ^2^ statistic and its degree of freedom. Values of *I*^2^ at 0 to 40% were considered possibly not important, 30 to 60% were considered moderate, 50 to 90% were considered substantial, and 75 to 100% were considered considerable heterogenic (42). If evidence of heterogeneity was found, meta-regression was carried out to explore the sources of heterogeneity. The moderators affecting the outcome variable DMI were the BW of the experimental cows, daily dose rate of Cr, duration of Cr supplementation or the experiment, type of Cr complex used (such as amino acids, propionate, or methionine), and the supplementation phase (BFP or AFP). The moderators affecting the outcome variables of milk production and composition (fat, protein, lactose, and SNF) were DIM, cow BW, Cr dose rate, duration of the experiment or Cr supplementation period, and the type of Cr complex.

Additionally, a multilevel meta-analysis was also carried out to evaluate the heterogeneity (43) at various levels. The variance distribution being as follows: level 1 = sampling variance, level 2 = effect sizes extracted from the same study, and level 3 = variance between studies. The multilevel meta-analysis was carried out using the metafor R package (version 3.0.2) (44). The variance distribution was evaluated by the R package dmetar (version 0.0.9000) (38).

The τ^2^ values of the models were compared with or without a moderator to evaluate the decrease in heterogeneity. Influence analysis for heterogeneity identification was carried out for the response of interest (DMI, milk production, fat, protein, lactose, and SNF) using the Baujat diagnostics. The Baujat et al. (45) diagnostics identified the respective contribution of each study.

### 2.3. Publication bias

A contour-enhanced funnel plot for each outcome was created to assess the risk of bias in the studies included in the meta-analysis. The standard error of the observed outcomes as predictor was used to check for funnel plot asymmetry. The symmetrical distribution of studies around the calculated MD indicated no risk of bias, while an asymmetrical distribution around MD was an indication of the potential risk of bias. The presence of bias was identified by Egger's test (46), and *P* < 0.05 indicated the presence of bias in the funnel plot. The analysis was carried out using R package metafor (version 3.0.2) (44).

## 3. Results

### 3.1. Dry matter intake

**Figure 2:**
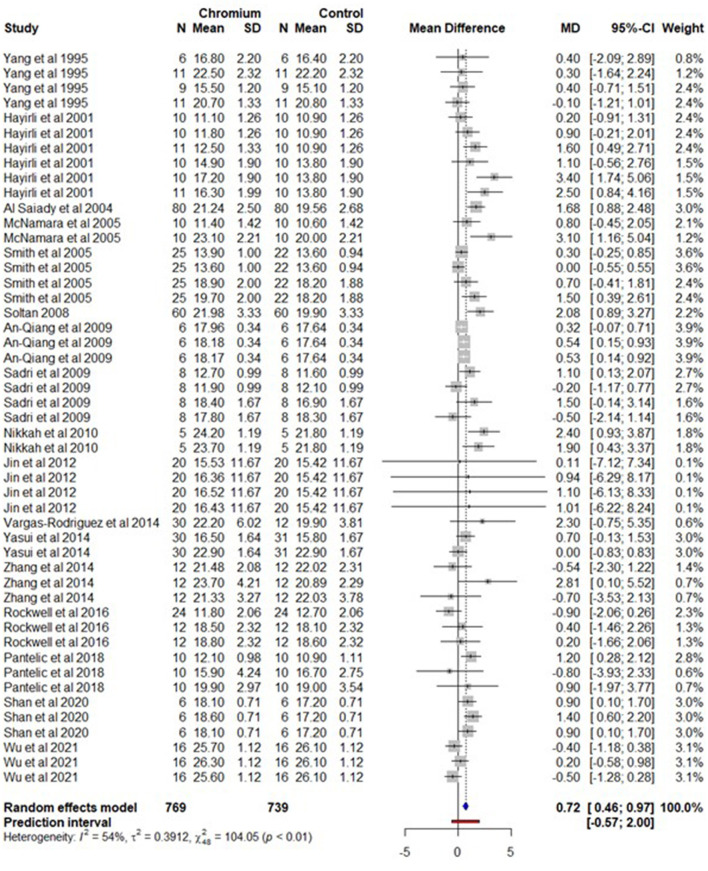
The forest plot of the random-effect meta-analysis for dry matter intake. The effect size was calculated as the mean difference (MD), and the solid vertical line represents the line of no effect or zero line. The dotted vertical line represents the average effect size for dry matter intake in cows supplemented with chromium. The negative value under the MD heading indicates a decrease in dry matter intake and vice versa. The black horizontal line represents the confidence interval for each study and the gray square represents weight or effect size of the corresponding study. The blue diamond represents the average effect size of the meta-analysis.

A total of 17 studies with 49 effect sizes and 769 observations for Cr supplementation and 739 observations for the control were included in the DMI meta-analysis (Supplementary Table 1). A forest plot (Figure 2) shows the observed outcomes and the estimate based on the random-effects model. The DMI was increased (*P* < 0.05) by 0.72 kg [95% confidence interval (CI), 0.46–0.97] in cows supplemented with Cr compared to those not supplemented. The heterogeneity for DMI was moderate (*I*^2^ = 54%, τ^2^ = 0.391, and Q statistic: χ^2^ = 104.05). The regression model indicates that DMI was significantly increased (*P* < 0.001) of 0.0009 kg/kg of BW (Table 1) (95% CI = 0.0004–0.001). The DMI also significantly increased (*P* > 0.05) by 0.0805 kg for an increase in each mg of Cr/cow/day (95% CI = 0.049–0.111). When the duration of Cr supplementation increases by 1 week, DMI significantly (*P* < 0.001) increased by 0.057 kg/day (95% CI = 0.030 – 0.084). The supplementation phase (BFP or AFP) is (*P* < 0.001) associated with an increase in DMI by 0.4582 and 0.853 kg/day respectively. The methionine and yeast forms of Cr increased DMI by 0.714 and 1.137 kg/day respectively. The DMI for MP and MP+PP cows increased (*P* < 0.001) by 0.620 and 2.137 kg/day respectively. The DMI was not influenced by Cr supplementation in PP cows (*P* > 0.05). The summary of the random effect model, multilevel random-effects model, and moderators is presented in Table 1. The funnel plot of the mean difference in DMI is plotted against the standard error of the experiment (Supplementary Figure 1). The symmetrical distribution of the weighted mean difference of all experiments around standard error indicates the absence of biasness in experiments selected for meta-analysis. The Egger's test was also nonsignificant (*P* = 0.135) with 95% CI = 0.0478–0.870.

**Table 1:** A summary of statistical models and moderators for the dry matter intake meta-analysis.

	**Estimate**	**Standard error**	***z***-**value**	***p***-**value**	**CI-LB**	**CI-UB**	**tau^2^**	**Reduction in tau^2^**	***n***
**Model**									
Random effect model	0.720	0.130	5.496	<0.001	0.460	0.970	0.391		49
Multilevel random effect	0.771	0.183	4.201	<0.001	0.402	1.140	0.400	2.302	49
**Moderator**									
BW	0.0009	0.000	3.908	<0.001	0.000	0.001	0.480	22.711	36
Dose	0.080	0.052	5.107	<0.001	0.050	0.113	0.454	16.061	49
Experimental duration	0.058	0.014	4.194	<0.001	0.031	0.085	0.533	36.266	49
**Parturition stage**									
AFP	0.853	0.161	5.293	<0.001	0.537	1.169	0.390	−0.358	49
BFP	0.458	0.220	2.087	<0.001	0.028	0.889			
**Cr-complex**									
Amino acids	0.020	0.480	0.428	0.668	0.736	1.147	0.334	−14.58	49
Methionine	0.714	0.186	3.850	<0.001	0.350	1.077			
Picolinate	0.430	0.319	1.346	0.178	0.196	1.057			
Propionate	0.518	0.320	1.615	0.106	0.110	1.146			
Yeast	1.137	0.308	3.690	0.002	0.533	1.742			
**Parity**									
MP	0.620	0.125	4.954	<0.001	0.375	0.866	0.275	−29.668	49
MP+PP	2.137	0.481	4.439	<0.001	1.193	3.081			
PP	0.371	0.482	0.770	0.440	0.573	1.316			

BW, body weight; AFP, after parturition; BFP, before parturition; PP, primiparous; MP, multiparous; CI-LB, confidence interval lower bound; CI-UB, confidence interval upper bound.

The multilevel meta-analytical model indicates that DMI was significantly higher (*P* < 0.001, MD = 0.771 kg/day (95% CI, 0.402–1.140) in cows supplemented with Cr. The multilevel variance (Supplementary Figure 2) indicates that the sampling variance (level 1) of the effect size was 36.9%. The variance between effect sizes extracted from the same study (level 2) was only 6.4%. However, the major portion of the variances (56.7%) was associated between studies (level 3). The influence analysis for the DMI meta-analysis is presented in Supplementary Figure 3.

### 3.2. Milk production

**Figure 3:**
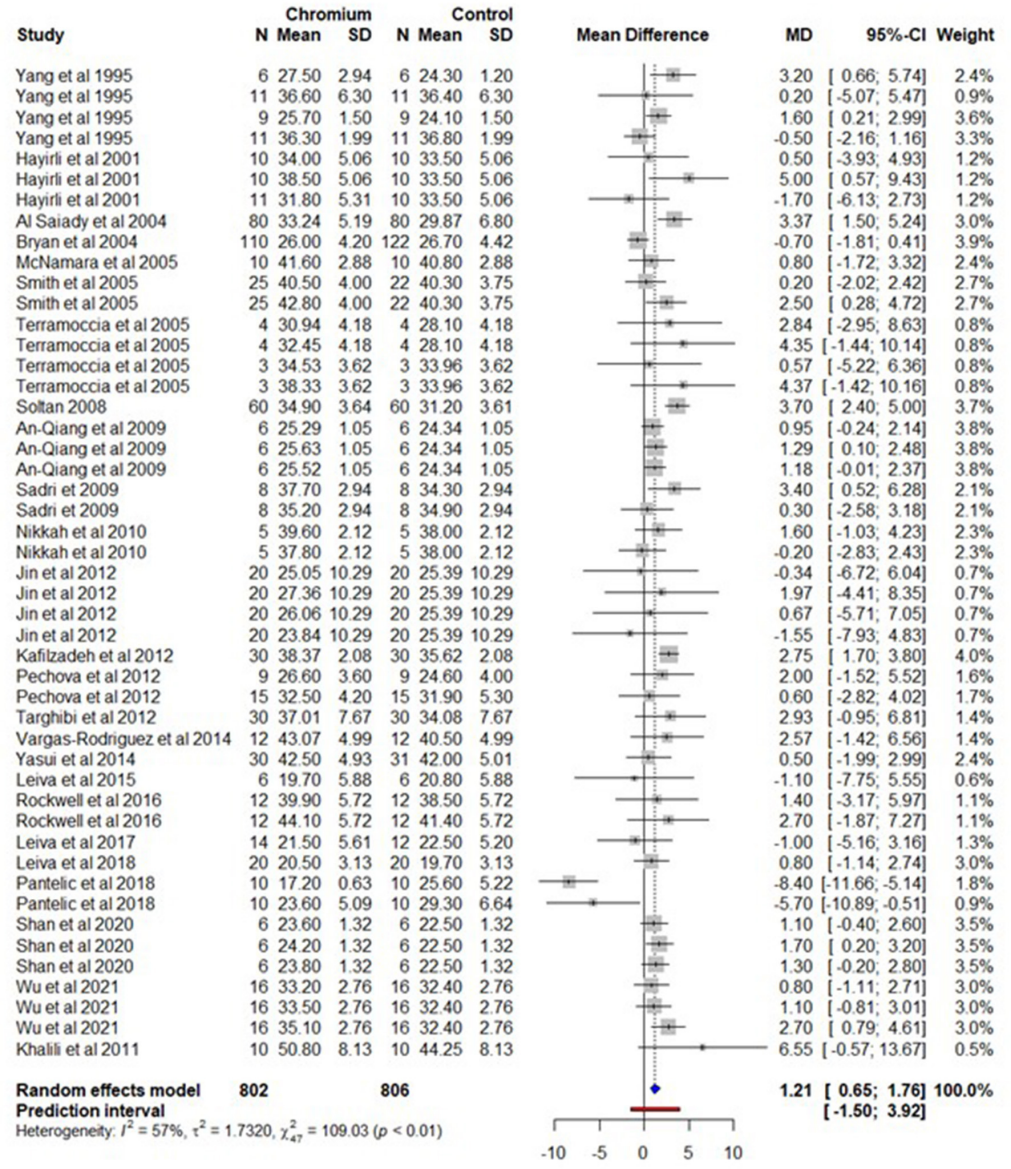
The forest plot of the random-effect meta-analysis for milk production. The effect size was calculated as the mean difference (MD) and the solid vertical line represents the line of no effect or zero line. The dotted vertical line represents the average effect size for milk production in cows supplemented with chromium and the negative value under the MD heading indicates a decrease in milk production and vice versa. The black horizontal line represents the confidence interval for each study and the gray square represents weight or effect size of the corresponding study. The blue diamond represents the average effect size of the meta-analysis.

For milk production, we selected 25 studies and 48 effect sizes with 802 cows for Cr supplementation and 806 cows for the control group (Supplementary Table 2). The observed outcomes and the estimate based on the random-effects model are shown in Figure 3. Milk production increased (*P* < 0.001) by 1.20 kg/day (95% CI, 0.65-1.76) with Cr supplementation. The heterogeneity for milk production was moderate (*I*^2^ = 57%, τ^2^ = 1.732, and *Q* statistic: χ^2^ = 100.03). The regression model indicates that milk production was significantly (*P* < 0.001) increased by 0.0023 kg/day (95% CI, 0.0015–0.0031) with each kg increase of BW. The milk production also increased (*P* = 0.004) by 0.1224 kg/day (95% CI, 0.0548–0.1900) with each mg/day increase in Cr supplementation. An increase in one week of the experiment/Cr supplementation duration resulted in a significant increase (*P* = 0.004) in milk production by 0.0676 kg/day (95% CI: 0.0129–0.1133). Similarly, the milk production increased (*P* = 0.045) by 0.0168 kg/day (95% CI, 0.0004–0.0332) with an increase in one DIM. The amino acid and methionine forms of Cr complexes increased milk production by 1.645 and 1.448 kg/day respectively. Similarly, parity had a significant (*P* = 0.004) effect on milk production. Milk production increased by 1.087 and 1.920 kg/day for multiparous (MP) and primiparous (PP) cows respectively. The summary of the random effect model, multilevel random-effects model, and moderators are presented in Table 2. The funnel plot of the mean difference in milk production is plotted against the standard error of the experiment in Supplementary Figure 4. Visual inspection implies that the symmetrical distribution of the weighted mean difference of all experiments around standard error indicates the absence of biasness in experiments selected for meta-analysis. The Egger's test was also nonsignificant (*P* = 0.585) with 95% CI = 0.367–2.527.

**Table 2:** A summary of statistical models and moderators for the milk production meta-analysis.

	**Estimate**	**Standard error**	***z***-**value**	***p***-**value**	**CI- LB**	**CI-UB**	**tau^2^**	**Reduction in tau^2^**	***n***
**Model**									
Random effect	1.210	0.283	4.251	<0.001	0.650	1.716	1.732		48
Multilevel random effect	1.186	0.413	2.872	0.006	0.355	2.017	1.675	−3.29	48
**Moderator**									
BW	0.002	0.000	5.394	<0.001	0.001	0.003	0.937	−45.90	34
Dose	0.122	0.035	3.550	<0.001	0.054	0.190	2.139	23.50	48
Experimental duration	0.068	0.023	2.901	0.004	0.021	0.113	2.563	47.98	48
Days in milk	0.017	0.008	2.009	0.045	0.000	0.033	3.011	73.85	48
**Cr-complex**									
Amino acids	1.645	0.830	1.982	0.047	0.018	3.271	2.172	25.40	48
Methionine	1.448	0.503	2.878	0.004	0.462	2.434			
Picolinate	1.140	0.921	1.238	0.215	0.664	2.944			
Propionate	0.760	0.745	1.019	0.307	0.700	2.221			
Yeast	0.149	0.761	0.195	0.845	1.344	1.641			
**Parity**									
MP	1.087	0.333	3.223	0.001	0.426	1.748	1.879	8.49	48
MP+PP	1.199	0.773	1.549	0.121	0.317	2.716			
PP	1.920	0.843	2.277	0.022	0.267	3.572			

BW, body weight; AFP, after parturition; BFP, before parturition; PP, primiparous; MP, multiparous; CI-LB, confidence interval lower bound; CI-UB, confidence interval upper bound.

The multilevel random effect meta-analytical model indicates that milk production was significantly higher (*P* = 0.001, 95% CI, 0.354–2.1017) with an effect size of 1.186 kg in cows supplemented with Cr. The multilevel variance indicates (Supplementary Figure 5) that the sampling variance (level 1) of the effect size was 30.4%. The variance between effect sizes extracted from the same study (level 2) was 0%. However, the major portion of the variances (69.6%) was associated between studies (level 3). The influence analysis for the DMI meta-analysis is presented in Supplementary Figure 6.

### 3.3. Milk protein

**Figure 4:**
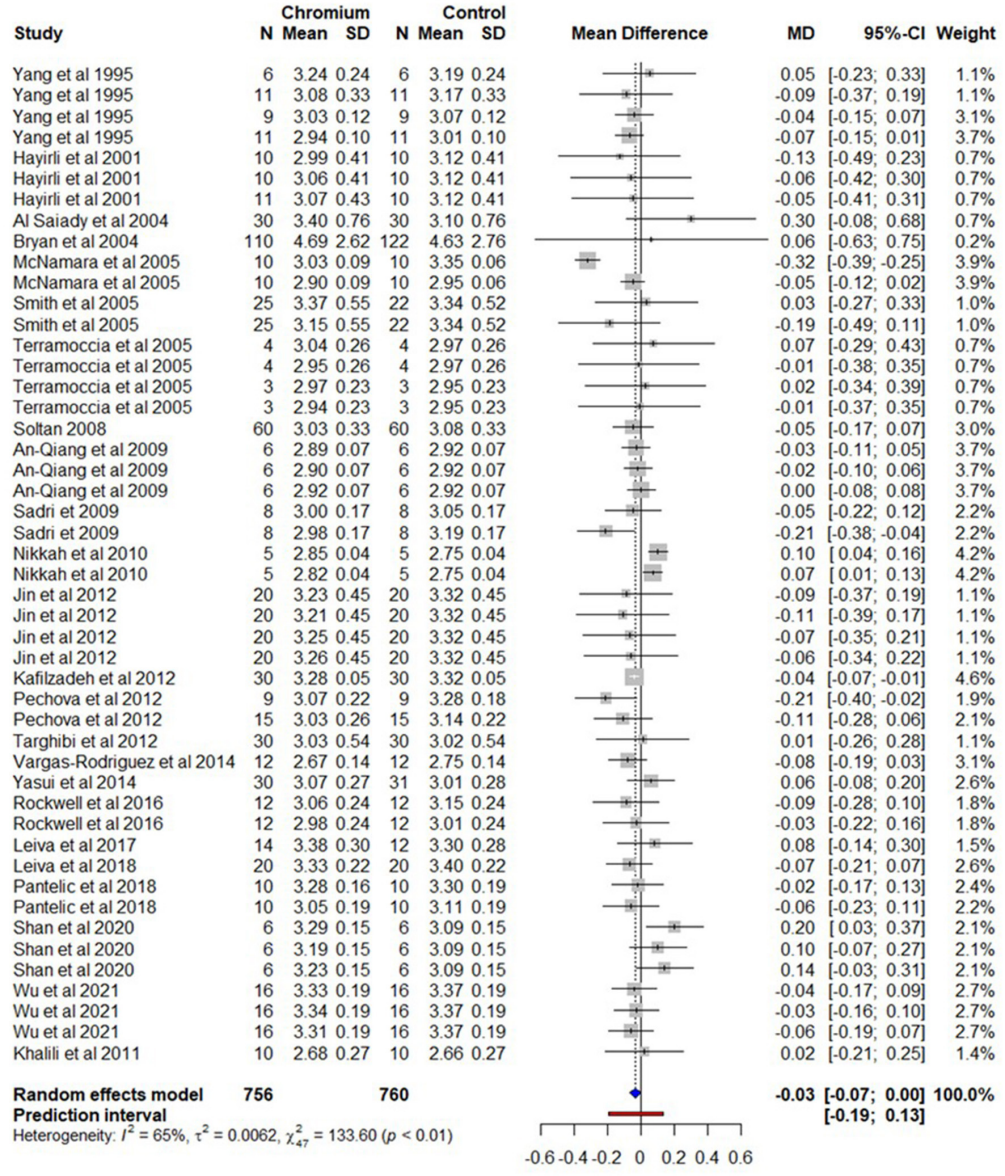
The forest plot of the random-effect meta-analysis for milk protein content. The effect size was calculated as the mean difference (MD), and the solid vertical line represents the line of no effect or zero line. The dotted vertical line represents the average effect size for milk protein content in cows supplemented with chromium. The negative value under the MD heading indicates a decrease in milk protein content and vice versa. The black horizontal line represents the confidence interval for each study and the gray square represents weight or effect size of the corresponding study. The blue diamond represents the average effect size of the meta-analysis.

A total of 24 studies with 48 effect sizes, with 756 cows for Cr supplemented and 760 cows for the control were included in the milk protein meta-analysis (Supplementary Table 3). The observed mean difference was−0.03% (*P* > 0.05) in milk protein content for the cows supplemented with Cr (Figure 4). The heterogeneity for milk protein content was moderate (*I*^2^ = 65%, τ^2^ = 0.0062, and Q statistic: χ^2^ = 133.60). The regression model indicates that BW, the dose of Cr (mg/day/cows), experiment duration/supplementation of Cr, DIM, and parity have no influence on effects size for milk protein content in cows supplemented with Cr. While the regression model indicates that milk protein decreased (*P* = 0.006) by −0.0868% with Cr complexes in propionate form.

The summary of the random effect model, multilevel random-effects model, and moderators are presented in Table 3. A funnel plot of the mean difference in milk protein content is plotted against the standard error of the experiment in Supplementary Figure 7. Visual inspection of the funnel plot indicates that symmetrical distribution of the weighted mean difference of all experiments around standard error indicates an absence of biasness in experiments selected for meta-analysis. The Egger's test was also nonsignificant (*P* = 0.682) with 95% CI = 0.1094–0.0214.

**Table 3:** A summary of statistical models and moderators for the milk protein meta-analysis.

	**Estimate**	**Standard error**	***z***-**value**	***p***-**value**	**CI-LB**	**CI-UB**	**tau^2^**	**Reduction in tau^2^**	***n***
**Model**									
Random effect model	−0.032	0.017	1.894	0.058	0.065	0.001	0.006		48
Multilevel random effect	−0.033	0.020	1.633	0.109	0.074	0.007	0.006	6.45	
**Moderator**									
BW	0	0	1.096	0.272	0.001	0	0.002	−67.74	33
Dose	−0.003	0.009	1.944	0.052	0.007	0	0.006	−3.23	48
Experimental duration	−0.002	0.001	1.516	0.129	0.004	0.0006	0.006	1.61	48
Days in milk	0.0006	0.0005	1.216	0.223	0.0004	0.001	0.006	8.06	48
**Cr-complex**									
Amino acids	−0.032	0.047	0.690	0.490	0.125	0.060	0.005	−17.74	
Methionine	−0.018	0.028	0.653	0.513	0.074	0.037			
Picolinate	−0.016	0.048	0.346	0.728	0.234	0.134			
Propionate	−0.086	0.032	2.712	0.006	0.149	0.024			
Yeast	0.081	0.048	1.695	0.091	0.012	0.176			
**Parity**									
MP	−0.038	0.019	1.950	0.051	0.076	0.0002	0.005	−6.45	48
MP+PP	0.023	0.043	0.528	0.528	0.062	0.108			
PP	−0.066	0.048	1.364	0.172	0.161	0.028			

BW, body weight; AFP, after parturition; BFP, before parturition; PP, primiparous; MP, multiparous; CI-LB, confidence interval lower bound; CI-UB, confidence interval upper bound.

The multilevel random effects meta-analytical model results revealed that milk protein was non-significant (*P* = 0.109, 95% CI, 0.0741–0.0077) in cows supplemented with Cr. The multilevel variance (Supplementary Figure 8) indicates that the sampling variance (level 1) of the effect size was 34.6%, the variance between effect sizes extracted from the same study (level 2) was 27.1%. The variance between studies (level 3) was 38.3%.

### 3.4. Milk fat

**Figure 5:**
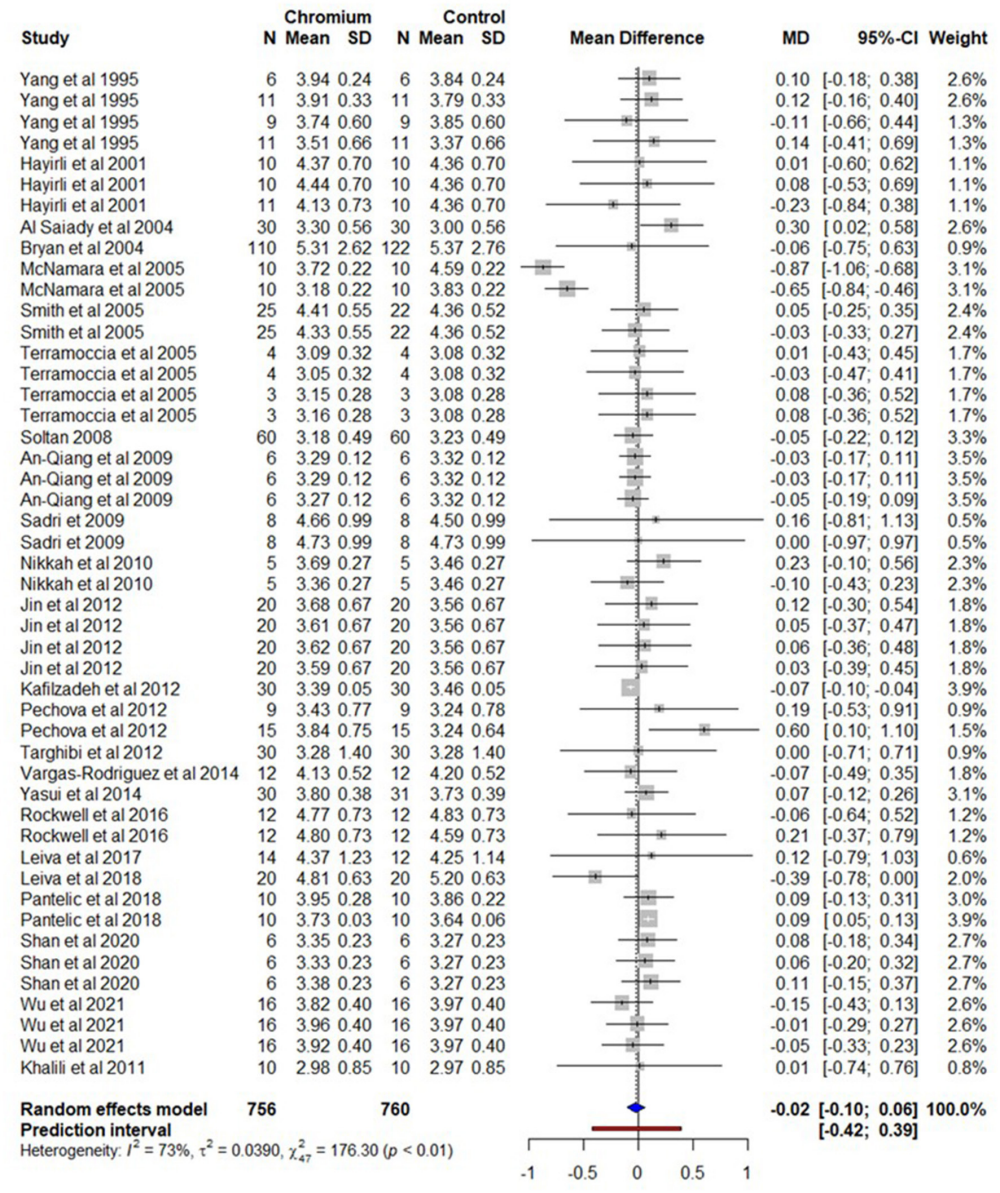
The forest plot of the random-effect meta-analysis for milk fat content. The effect size was calculated as the mean difference (MD), and the solid vertical line represents the line of no effect or zero line. The dotted vertical line represents the average effect size for milk fat content in cows supplemented with chromium. The negative value under the MD heading indicates a decrease in milk fat content and vice versa. The black horizontal line represents the confidence interval for each study and the gray square represents weight or effect size of the corresponding study. The blue diamond represents the average effect size of the meta-analysis.

A total of 24 studies with 48 effect sizes were included in the meta-analysis for milk fat content. The total number of cows for Cr and the control were 756 and 760 respectively (Supplementary Table 4). A forest plot (Figure 5) shows the observed outcomes and the estimate based on the random-effects model. The observed mean difference was a non-significant (*P* > 0.05) decrease in milk fat content in cows supplemented with Cr. The heterogeneity for milk fat content was substantial (*I*^2^ = 73%, τ^2^ = 0.390, and Q statistic: χ^2^ = 176.30).

The results of the regression model showed that factors including BW, Cr dose (mg/day/cow), experiment or supplementation duration, DIM, and parity had no significant impact (*P* > 0.05) on the fat content of milk produced by cows supplemented with Cr. Cr propionate complex supplementation had a negative influence on milk fat content with a 0.216% decrease (*P* = 0.003).

The summary of the random effect model, multilevel random-effects model, and moderators are presented in Table 4. A funnel plot of the mean difference in milk fat content is plotted against the standard error of the experiment in Supplementary Figure 9. Visual inspection of the symmetrical distribution of the weighted mean difference of all experiments around standard error shows the absence of biasness in experiments selected for meta-analysis. The Egger's test was also not significant (*P* = 0.216) with 95% CI = 0.2553–0.0500.

**Table 4:** A summary of statistical models and moderators for the milk fat meta-analysis.

	**Estimate**	**Standard error**	***z-*** **value**	***p-*** **value**	**CI–LB**	**CI–UB**	**tau^2^**	**Reduction in tau^2^**	***n***
**Model**									
Random effect model	−0.02	0.0392	0.488	0.625	0.096	0.0577	0.039		48
Multilevel random effect	−0.0146	0.052	0.277	0.782	0.1203	0.0921	0.043	10.51	
**Moderator**									
BW									33
Dose	−0.0043	0.0042	1.012	0.311	0.0125	0.004	0.038	−3.59	48
Experimental duration	−0.0013	0.0032	0.417	0.676	0.0076	0.005	0.039	0.00	48
Days in milk	−0.0002	0.0011	0.148	0.882	0.0023	0.0019	0.04	2.31	48
**Cr-complex**									
Amino acids	0.0591	0.094	0.624	0.532	0.1267	0.2444	0.028	−29.23	
Methionine	−0.0228	0.067	0.34	0.733	0.5141	0.1085			
Picolinate	−0.0367	0.104	0.351	0.724	0.2409	0.1676			
Propionate	−0.2169	0.073	2.954	0.003	0.3608	0.0730			
Yeast	0.1156	0.826	1.411	0.158	0.0453	0.2784			
**Parity**									
MP	−0.0346	0.046	0.75	0.452	0.1248	0.0557	0.04	3.08	48
MP + PP	−0.005	0.121	0.041	0.967	0.2435	0.2334			
PP	0.0464	0.1	0.463	0.643	0.1499	0.2427			

BW, body weight; AFP, after parturition; BFP, before parturition; PP, primiparous; MP, multiparous; CI-LB, confidence interval lower bound; CI-UB, confidence interval upper bound.

The multilevel meta-analytical model indicates that milk fat content was not influenced (*P* = 0.782) by Cr supplementation and the estimate for milk fat content was −0.0146 with 95% CI = 0.1203–0.0912. The multilevel variance (Supplementary Figure 10) indicates that the sampling variance (level 1) of the effect size was 15.7%, the variance between effects sizes extracted from the same study (level 2) was 0%. However, the major portion of the variances (84.3%) was associated among studies (level 3).

### 3.5. Milk lactose

**Figure 6:**
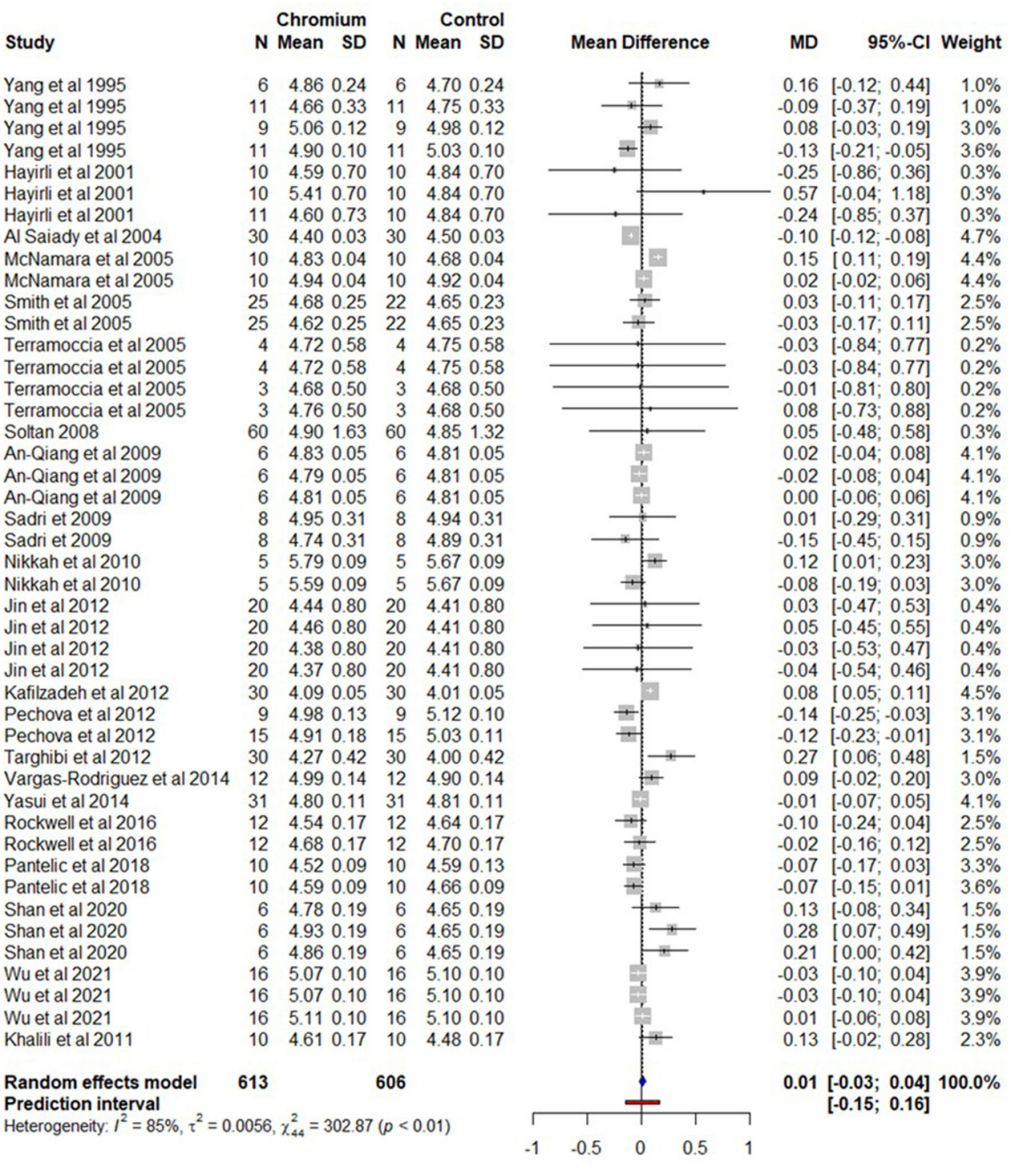
The forest plot of the random-effect meta-analysis for milk lactose content. The effect size was calculated as the mean difference (MD), and the solid vertical line represents the line of no effect or zero line. The dotted vertical line represents the average effect size for milk lactose content in cows supplemented with chromium. The negative value under the MD heading indicates a decrease in milk lactose content and vice versa. The black horizontal line represents the confidence interval for each study and the gray square represents weight or effect size of the corresponding study. The blue diamond represents the average effect size of the meta-analysis.

For milk lactose content, a total of 21 studies with 45 effect sizes were included in the meta-analysis, there were 613 cows for Cr supplemented and 606 cows for control (Supplementary Table 5). A forest plot (Figure 6) shows the observed outcomes and the estimate based on the random-effects model. The observed mean difference indicates that Cr supplementation had no effects (*P* > 0.05) on milk lactose content in cows. The heterogeneity for milk lactose content was substantial (*I*^2^ = 85%, τ^2^ = 0.0056, and Q statistic: χ^2^ = 302.87). The regression model indicates that effect size was not influenced by moderators including BW, the dose of Cr, experimental duration/supplementation of Cr, and Cr complex forms. While milk lactose content was increased (*P* = 0.042) by 0.0010% with increase in each day of DIM. The summary of the random effect model, multilevel random-effects model, and moderators are presented in Table 5. A funnel plot of the mean difference in milk lactose content plotted against the standard error of the experiment is shown in Supplementary Figure 11. By the visual inspection of the funnel plot, the symmetrical distribution of the weighted mean difference of all experiments around standard error indicates the absence of biasness in experiments selected for meta-analysis. The Egger's test was also nonsignificant (*P* = 0.407) with 95% CI = 0.0531–0.0381.

**Table 5:** A summary of statistical models and moderators for milk lactose meta-analysis.

	**Estimate**	**Standard error**	***z-*** **value**	***p-*** **value**	**CI–LB**	**CI–UB**	**tau^2^**	**Reduction in tau^2^**	***n***
**Model**									
Random effect model	0.0100	0.0162	0.3921	0.695	−0.0255	0.0380	0.0056		45
Multilevel random effect	0.0096	0.0210	0.4797	0.634	0.0308	0.0501	0.0064	14.29	
**Moderator**									
BW	0.0000	0.0000	0.4780	0.633	0.0001	0.0000	0.0032	−42.86	30
Dose	0.0006	0.0019	0.3020	0.763	0.0032	0.0040	0.0056	0.00	
Experimental duration	0.0004	0.0015	0.2380	0.812	0.0026	0.0030	0.0056	0.00	
Days in milk	0.0054	0.0001	2.0290	0.042	0.0000	0.0020	0.0054	−3.57	45
**Cr-complex**									
Amino acids	−0.0173	0.0523	0.3310	0.740	0.1197	0.0851	0.0051	−8.93	45
Methionine	0.0264	0.0272	0.9710	0.332	0.0269	0.0798			
Picolinate	0.0000	0.0445	0.0000	0.858	0.0873	0.0873			
Propionate	0.0311	0.0333	0.9350	1.000	0.0341	0.0963			
Yeast	−0.0031	0.0394	0.0780	0.350	0.0804	0.7420			
**Parity**									
MP	0.0034	0.0183	0.1878	0.851	0.0324	0.0393	0.0059	5.36	45
MP + PP	0.0436	0.0540	0.8063	0.420	0.0624	0.1495			
PP	−0.0031	0.0551	0.0566	0.955	0.1112	0.1049			

BW, body weight; AFP, after parturition; BFP, before parturition; PP, primiparous; MP, multiparous; CI-LB, confidence interval lower bound; CI-UB, confidence interval upper bound.

The multilevel random effects of the meta-analytical model revealed that milk lactose content was not significant (*P* = 0.633) in cows supplemented with Cr and the observed MD was 0.0096 with 95% CI, 0.0308–0.0501). The multilevel variance (Supplementary Figure 12) indicates that the sampling variance (level 1) of the effect size was 18.0%. The variance between effect sizes extracted from the same study (level 2) was 36.0%. However, the variance among studies (level 3) was 46.0%.

### 3.6. Solids-not-fat

**Figure 7:**
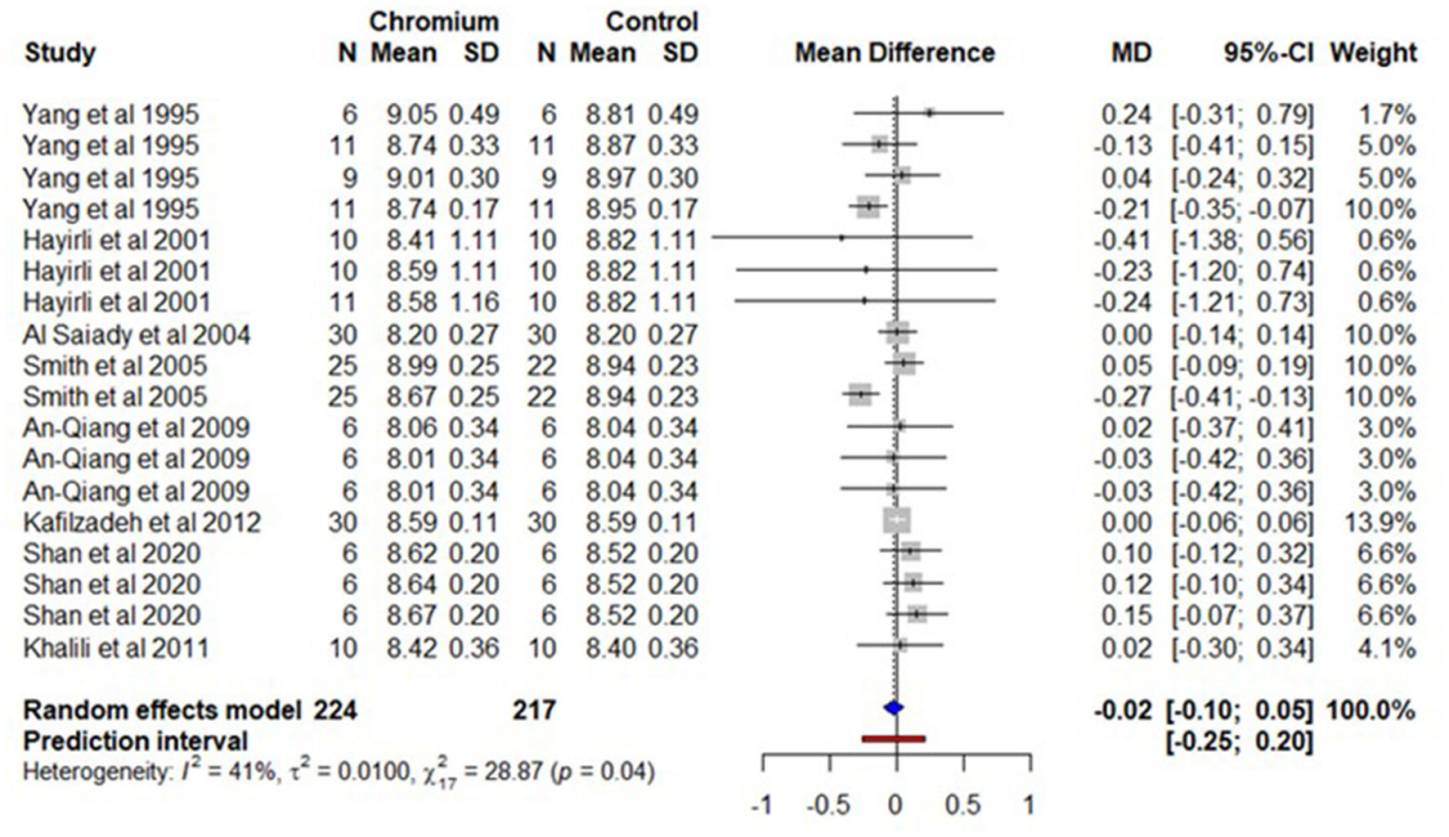
The forest plot of the random-effect meta-analysis for solid-not-fat content. The effect size was calculated as the mean difference (MD), and the solid vertical line represents the line of no effect or zero line. The dotted vertical line represents the average effect size for solid-not-fat content in cows supplemented with chromium. The negative value under the MD heading indicates a decrease in solid-not-fat content and vice versa. The black horizontal line represents the confidence interval for each study and the gray square represents weight or effect size of the corresponding study. The blue diamond represents the average effect size of the meta-analysis.

For SNF, only 8 studies with 18 effect sizes met the inclusion criteria. The total number of cows for Cr supplementation was 224 and 217 for the control. The raw data are provided in Supplementary Table 6. A forest plot (Figure 7) shows the observed outcomes and the estimate based on the random-effects model. The observed mean difference was not significantly different (*P* = 0.523) between Cr supplementation and the control. The heterogeneity for SNF was moderate (*I*^2^ = 41%, τ^2^ = 0.010, and Q statistic: χ^2^ = 28.87). The regression model indicates that SNF was not influenced (*P* > 0.05) by moderators including BW, the dose of Cr supplementation, the duration of Cr supplementation/the duration of the experiment, Cr complex forms, and parity. The summary of the random effect model, multilevel random-effects model, and moderators are presented in Table 6. A funnel plot of the mean difference in SNF content was plotted against the standard error of the experiment and is shown in Supplementary Figure 13. Visual inspection of the symmetrical of the weighted mean difference of all experiments around standard error indicates the absence of biasness in experiments selected for meta-analysis. The Egger's test was also non-significant (*P* = 0.962) with 95% CI = 0.1615–0.107.

**Table 6:** A summary of statistical models and moderators for solids-not-fat meta-analysis.

	**Estimate**	**Standard error**	***z***-**value**	***p***-**value**	**CI–LB**	**CI–UB**	**tau^2^**	**Reduction in tau^2^**	***n***
**Model**									
Random effect model	−0.0247	0.038	0.638	0.523	0.1004	0.0511	0.0100		18
Multilevel random effect	−0.0250	0.040	0.619	0.543	0.1100	0.0601	0.0103	3.00	18
**Moderator**									
BW	−0.0001	0.000	1.582	0.113	0.0002	0.0000	0.0092	−8.00	14
Dose	−0.0055	0.005	1.060	0.289	0.0156	0.0046	0.0088	−12.00	18
Experimental duration	−0.0025	0.003	0.879	0.379	0.0082	0.0031	0.0089	−11.00	18
Days in milk	0.0011	0.001	1.398	0.162	0.0005	0.0027	0.0088	−12.00	18
**Cr-complex**									
Amino acids	−0.1032	0.080	1.291	0.196	0.2590	0.2590	0.0084	−16.00	18
Methionine	−0.0647	0.058	1.118	0.263	0.1780	0.1780			
Picolinate	−0.0133	0.126	0.106	0.915	0.2602	0.2602			
Yeast	0.0807	0.068	1.187	0.234	0.0525	0.0525			
**Parity**									
MP	−0.0327	0.041	0.808	0.419	0.1120	0.0466	0.0104	4.00	18
PP	0.0903	0.151	0.599	0.549	0.2053	0.3859			

BW, body weight; AFP, after parturition; BFP, before parturition; PP, primiparous; MP, multiparous; CI-LB, confidence interval lower bound; CI-UB, confidence interval upper bound.

The multilevel meta-analytical model indicates that SNF was not significantly different (*P* = 0.543) between cows supplemented with Cr supplementation and the control and the estimate for SNF was−0.025%, with 95% CI, 0.110 – 0.060. The multilevel variance (Supplementary Figure 14) indicates that the sampling variance (level 1) of the effect size was 47.5%. The variance between effect sizes extracted from the same study (level 2) was only 45.7% and variances associated among studies (level 3) was 6.8%.

## 4. Discussion

The present work suggests that supplementation of Cr improves DMI and effect size and heterogeneity for the multilevel random-effects model (0.77 kg, τ^2^ = 0.40) and random-effects model (0.72 kg, τ^2^ = 0.39) were similar. The tau τ^2^ in the random-effects model without moderators was 0.39 (SE = 0.130), including the significant moderators in our model. We observed that BW, dose, and experiment duration did not decrease in τ^2^. Additionally, these moderators had minimal effects on effect size (BW = 0.0009, dose = 0.080, and experiment duratio*n* = 0.058). The increase in τ^2^value for BW is due to a lesser number of studies (*n* = 36) as compared to the random-effects model without any moderator (*n* = 49). There were some studies that have not provided the initial BW of the cows in the experiment. Due to these missing covariates, we analyzed our data as individual moderators. In the multiple meta-regression model, the R package metafor (44) automatically drops missing variables completely from the analysis. Intuitively, we would expect that the estimate of τ^2^ in the meta-regression model must be lower (or at least, no larger) than the estimate of τ^2^ from the random-effects model. However, we also observed an increase in τ^2^ for the dose of Cr and experimental duration/supplementation duration. In classical regression, adding a predictor can only decrease the residual variance. But in a multilevel model, adding an individual predictor can sometimes make the group-level variance go up. This can happen when the individual-level predictor is negatively correlated with the group coefficient (47). The τ^2^ value for the parturition stage (AFP and BFP), Cr complexes, and parity was decreased by −0.4%, −14.6%, and −29.7% respectively. The increase in DMI with Cr supplementation was greater for AFP (0.853 kg) than for BFP (0.458). These differences in DMI of dairy cows could be attributed to different physiological stages of the cows (7). The DMI was not influenced by Cr supplementation in PP cows, which might be associated with greater stress in PP cows as compared to MP (48). The increase in DMI during BFP and AFP could be associated with a decrease in non-esterified fatty acids (NEFA) by Cr supplementation (26). As a consequence, an increase in DMI *via* reversing the lipostatic mechanism as high circulating NEFA concentrations depress feed intake (32). The DMI tended to increase during the prepartum period (35). Similarly, several studies have shown that supplementing with Cr increases DMI during the postpartum period (15, 29). It is unknown whether the effects of Cr supplementation on DMI are directly or indirectly mediated through other mechanisms in metabolism, or simply a result of generally increased milk yield with Cr supplementation (22). Therefore, it is difficult to determine whether the positive effects of Cr administration on performance responses were merely due to alleviation of a deficiency or some other factors related to the physiological mode of action of Cr.

Chromium supplementation increased milk production by 1.21 kg, and the effects size for the multilevel random-effects model (1.186 kg) and random-effects model (1.210 kg) were similar. This result enhances our confidence in the application of Cr supplement for the promotion of milk production. The τ^2^ in the random-effects model without moderator was 1.732 (SE = 0.283). For multilevel random-effects model τ^2^ = 3.3% decreases as compared to random-effects model. The DMI is an important factor that affects milk production, as the cow needs to consume sufficient nutrients to support milk synthesis. The increased milk yield can probably be explained by higher DMI and efficiency of energy utilization (49). After incorporating the significant moderating variables into our model, we found that a decrease in BW reduced the overall heterogeneity by 45.9. There was a slight impact on the effect size, with a 0.002 kg increase in milk production for per kg increase in BW of Cr-supplemented cows. Additionally, an increase of 1 mg in Cr dosage led to a 0.122 kg increase in milk production. The effect of experiment duration (estimate = 0.068) and DIM (estimate = 0.017) on milk production was minimal. Among Cr complexes only amino acid and methionine have significant effects on milk production and the MD were 1.645 and 1.448 kg respectively. The increase in milk production could be associated with an increase in lactose content of the milk, the major regulator of milk volume (50). It is known that the mammary gland consumes circulating glucose to synthesize milk lactose in mammary epithelial cells (51). Supplementation of Cr might increase glucose uptake by the cells (7). However, the underlying mechanism of the increase in milk lactose content needs further investigation as our meta-analytical results suggest that no differences were observed for lactose content of milk in Cr-supplemented cows. Additionally, the lactose yield was also calculated and compared using analysis of variance. The control group produced 1549 g lactose/day/cow, while the Cr-supplemented group produced 1635 g/day/cow (*P* = 0.2704). Despite the higher lactose yield in the Cr-supplemented group, it was linked to the increased milk production. The raw data show that control cows produced 32.63 kg of milk per day per cow, while the Cr-supplemented cows produced 34.25 kg. This translates to a 4.96% increase in milk and similarly higher lactose yield (5.55%) in the Cr-supplemented group.

Milk protein content was not influenced by Cr, and the effects size and heterogeneity for the multilevel random-effects model (MD = −0.0332%, τ^2^ = 0.0066) and random effect model (−0.0324, τ^2^ = 0.0062) were similar. With moderators included in our model, we observed that BW decreases τ^2^ = −67.7% of total heterogeneity, additionally, picolinate (Cr complexes) and parity decreased −17.7% and −6.5% heterogeneity respectively. Milk fat content, milk lactose, and SNF are not influenced by Cr supplementation. Regarding heterogeneity, we observed our *I*^2^ was > 50% in all meta-analytical data sets. Several studies have reported that the supplementation of Cr had no effects on fat, protein, and lactose percentage of dairy cow milk (15, 33, 36). These results suggest that it is almost impossible to improve milk quality by supplementing Cr to cows in dairying practice.

## 5. Conclusion

Chromium supplementation increases DMI and the response to supplementation is less before parturition (estimate = 0.458 kg) than after parturition (estimate = 0.853 kg). In terms of parity, DMI was 0.620 kg higher with Cr supplementation for multiparous cows compared to the unsupplemented control. For the milk production meta-analysis, the observed mean difference indicates that milk production increases by 1.20 kg with Cr supplementation. For multiparous and primiparous cows, milk production increased by 1.087 kg and 1.92 kg, respectively. The milk protein content, milk fat content, milk lactose content, and solids-not-fat are not influenced by Cr supplementation. Further research is warranted to evaluate the effects of Cr propionate on milk fat content and milk protein. The heterogeneity data indicate both milk fat and protein contents decrease with the supplementation of Cr propionate.

## Data availability statement

The original contributions presented in the study are included in the article/Supplementary material, further inquiries can be directed to the corresponding authors.

## Author contributions

MM conceived and planned the study and collected all data. MM, DR, and XS analyzed, interpreted data, prepared the tables, prepared the figures, and wrote the manuscript. XS and XZ acquired funding and revised the manuscript. All authors contributed to the article and approved the submitted version.

## Supplementary material

Supplementary Figure 1 A contour-enhanced funnel plot. The symmetrical distribution of studies around the mean difference (MD; x-axis) indicates that there were no publication biases in the studies included in the meta-analysis. The Egger's test was also not significant (*P* > 0.05). The vertical dotted line indicates the weighed MD effect size for dry matter intake in chromium-supplemented cows.

Supplementary Figure 2 The variance components identified by multilevel random effects in the dry matter intake meta-analysis. Level 1 = sampling variance of the extracted effect size, level 2 = variance between effects sizes extracted from the same study, level 3 = variance among the studies selected for meta-analysis.

Supplementary Figure 3 Graphical presentation of the influence analysis of the dry matter intake meta-analysis.

Supplementary Figure 4 A contour-enhanced funnel plot. The symmetrical distribution of studies around the mean difference (MD; x-axis) indicates that there were no publication biases in the studies included in the meta-analysis. The Egger's test was also not significant (*P* > 0.05). The vertical dotted line indicates the weighed MD effect size for milk production in chromium-supplemented cows.

Supplementary Figure 5 The variance components identified by multilevel random effects in the milk production meta-analysis. Level 1 = sampling variance of the extracted effect size, level 2 = variance between effects sizes extracted from the same study, level 3 = variance among the studies selected for meta-analysis.

Supplementary Figure 6 Graphical presentation of the influence analysis of the milk production meta-analysis.

Supplementary Figure 7 A contour-enhanced funnel plot. The symmetrical distribution of studies around the mean difference (MD; x-axis) indicates that there were no publication biases in the studies included in the meta-analysis for milk protein content. The Egger's test was also not significant (*P* > 0.05). The vertical dotted line indicates the weighed MD effect size for milk protein content in chromium-supplemented cows.

Supplementary Figure 8 The variance components identified by multilevel random effects in the milk protein content meta-analysis. Level 1 = sampling variance of the extracted effect size, level 2 = variance between effect sizes extracted from the same study, level 3 = variance among the studies selected for meta-analysis.

Supplementary Figure 9 A contour-enhanced funnel plot. The symmetrical distribution of studies around the mean difference (MD; x-axis) indicates that there were no publication biases in the studies included in the meta-analysis for milk fat content. The Egger's test was also not significant (*P* > 0.05). The vertical dotted line indicates the weighed MD effect size for milk fat content in chromium-supplemented cows.

Supplementary Figure 10 The variance components identified by multilevel random effects in the milk fat content meta-analysis. Level 1 = sampling variance of the extracted effect size, level 2 = variance between effects sizes extracted from the same study, level 3 = variance among the studies selected for meta-analysis.

Supplementary Figure 11 A contour-enhanced funnel plot. The symmetrical distribution of studies around the mean difference (MD; x-axis) indicates that there were no publication biases in the studies included in the meta-analysis for milk lactose content. The Egger's test was also not significant (*P* > 0.05). The vertical dotted line indicates the weighed MD effect size for milk lactose content in chromium-supplemented cows.

Supplementary Figure 12 The variance components identified by multilevel random effects in the milk lactose content meta-analysis. Level 1 = sampling variance of the extracted effect size, level 2 = variance between effects sizes extracted from the same study, level 3 = variance among the studies selected for meta-analysis.

Supplementary Figure 13 A contour-enhanced funnel plot. The symmetrical distribution of studies around the mean difference (MD; x-axis) indicates that there were no publication biases in the studies included in the meta-analysis for solid-not-fat content. The Egger's test was also not significant (*P* > 0.05). The vertical dotted line indicates the weighed MD effect size for solid-not-fat content in chromium-supplemented cows.

Supplementary Figure 14 The variance components identified by multilevel random effects in the solid-not-fat content meta-analysis. Level 1 = sampling variance of the extracted effect size, level 2 = variance between effects sizes extracted from the same study, level 3 = variance among the studies selected for meta-analysis.
