# Supplementary figures and images for: Effects of dietary chromium supplementation on dry matter intake and milk production and composition in lactating dairy cows: A meta-analysis
Source: Front Vet Sci. 2023 Mar 16;10:1076777. doi: 10.3389/fvets.2023.1076777 (PMC10062059; doi:10.3389/fvets.2023.1076777)

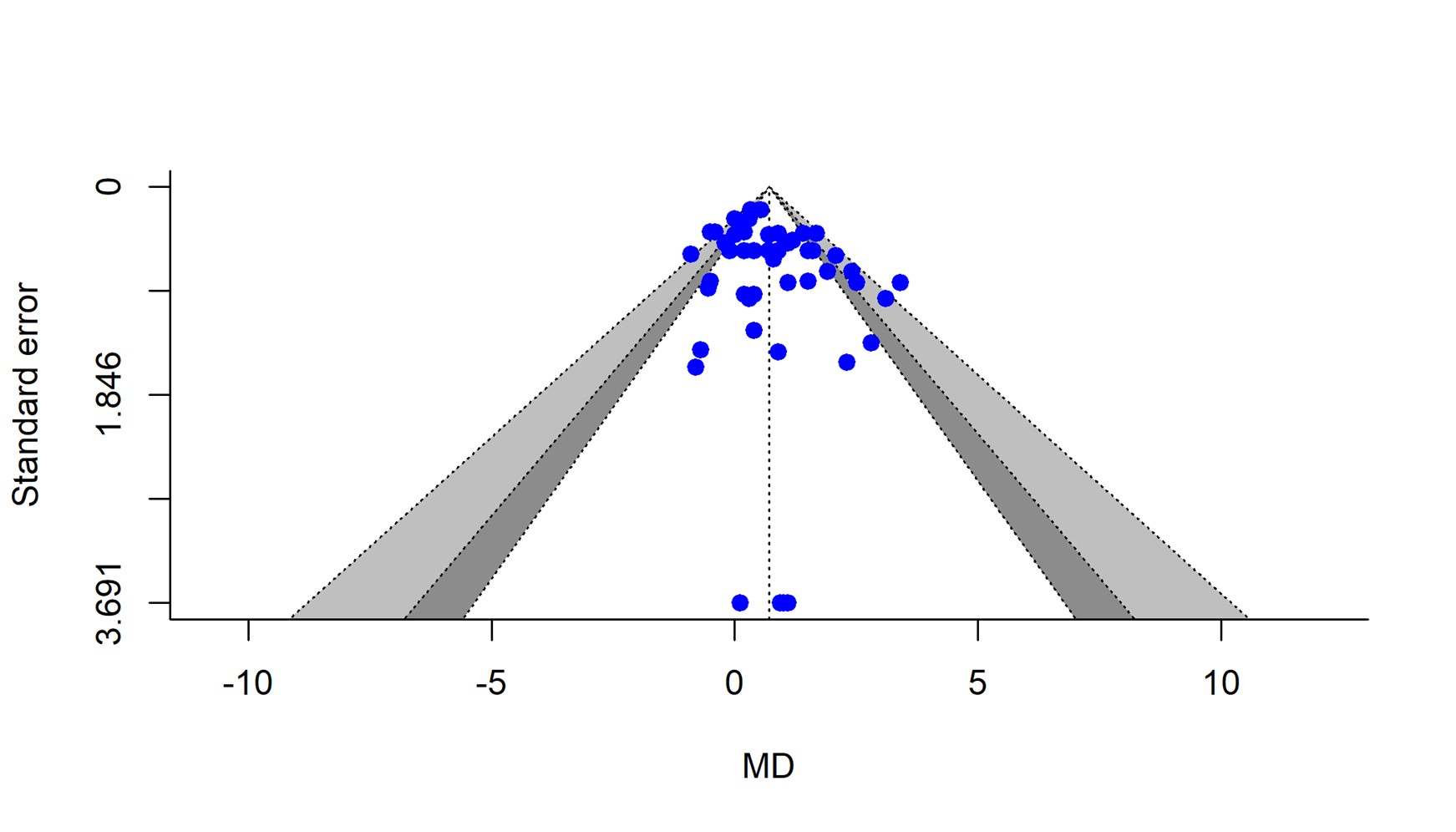

Supplement: Supplementary Figure 1 — A contour-enhanced funnel plot. The symmetrical distribution of studies around the mean difference (MD; x-axis) indicates that there were no publication biases in the studies included in the meta-analysis. The Egger's test was also not significant (P > 0.05). The vertical dotted line indicates the weighed MD effect size for dry matter intake in chromium-supplemented cows. [file Image_1.JPEG]

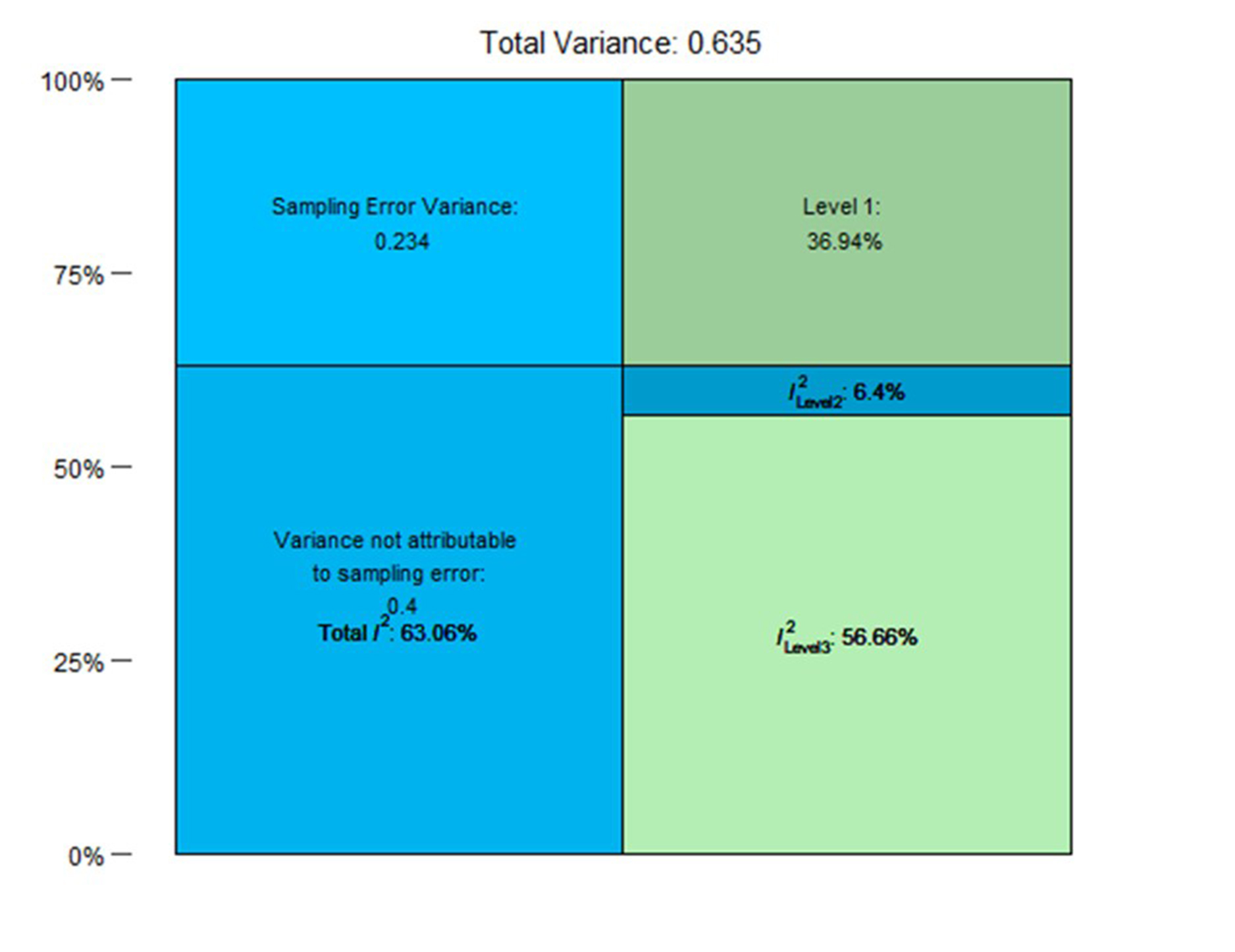

Supplement: Supplementary Figure 2 — The variance components identified by multilevel random effects in the dry matter intake meta-analysis. Level 1 = sampling variance of the extracted effect size, level 2 = variance between effects sizes extracted from the same study, level 3 = variance among the studies selected for meta-analysis. [file Image_2.JPEG]

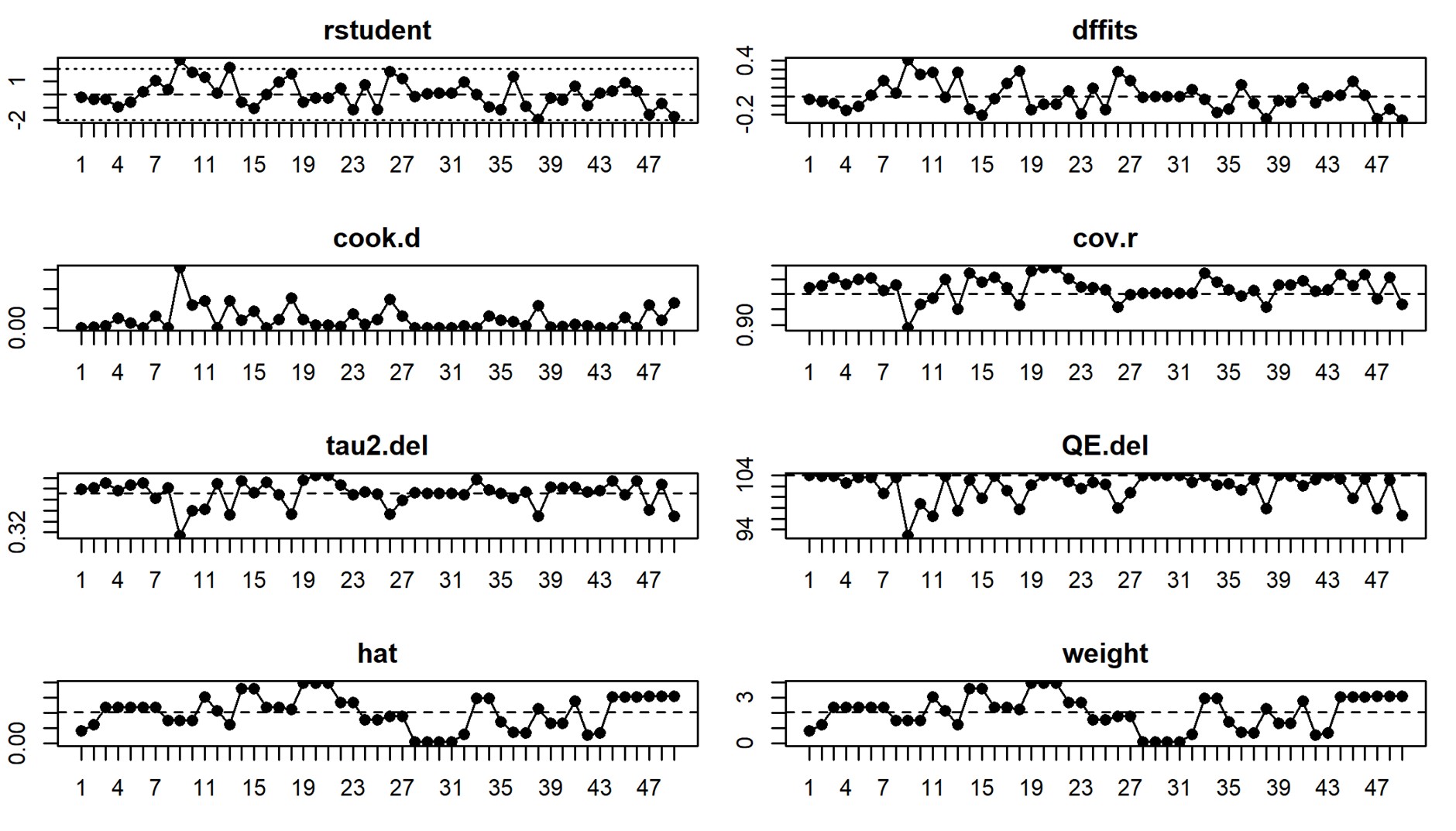

Supplement: Supplementary Figure 3 — Graphical presentation of the influence analysis of the dry matter intake meta-analysis. [file Image_3.JPEG]

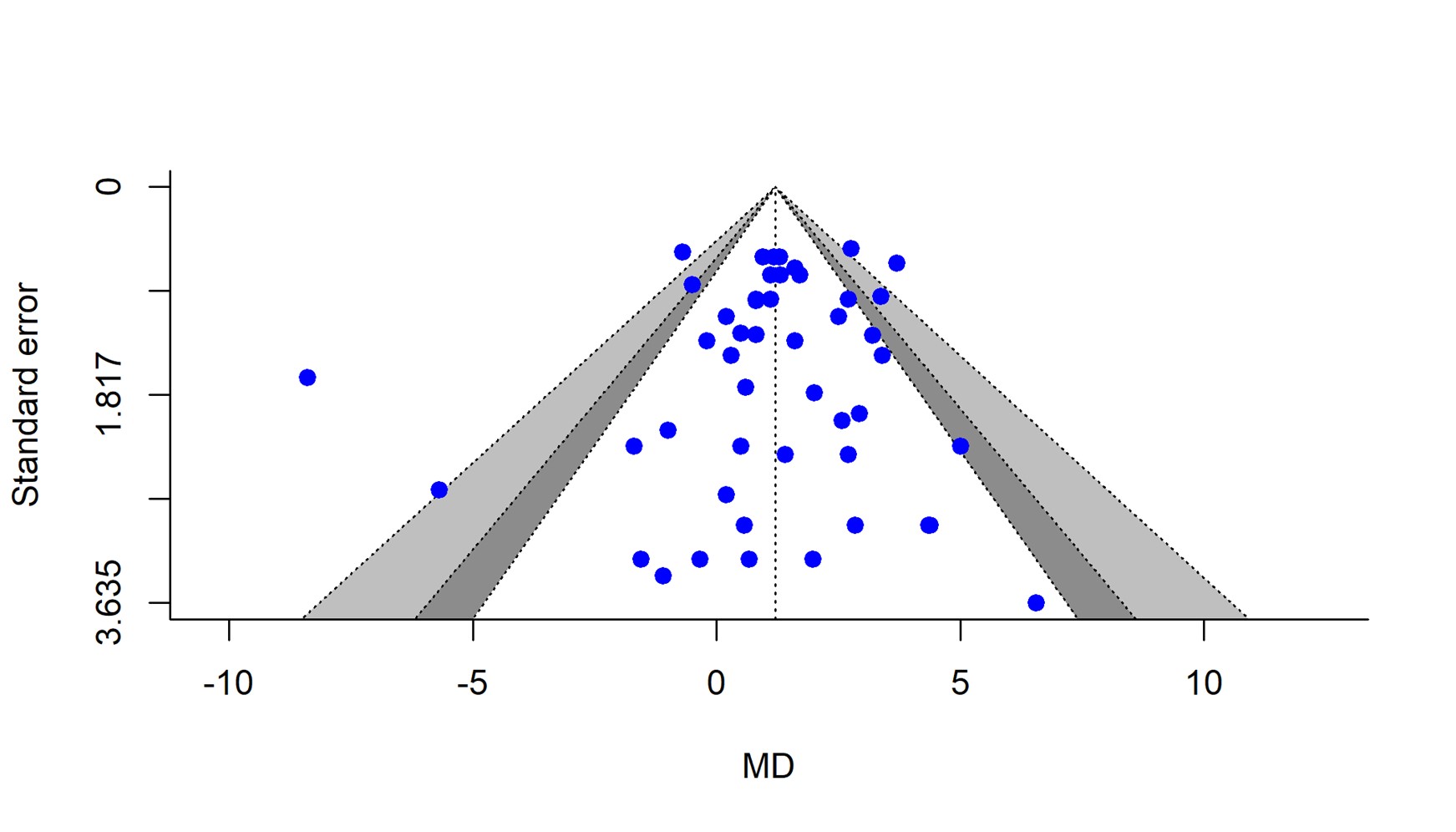

Supplement: Supplementary Figure 4 — A contour-enhanced funnel plot. The symmetrical distribution of studies around the mean difference (MD; x-axis) indicates that there were no publication biases in the studies included in the meta-analysis. The Egger's test was also not significant (P > 0.05). The vertical dotted line indicates the weighed MD effect size for milk production in chromium-supplemented cows. [file Image_4.JPEG]

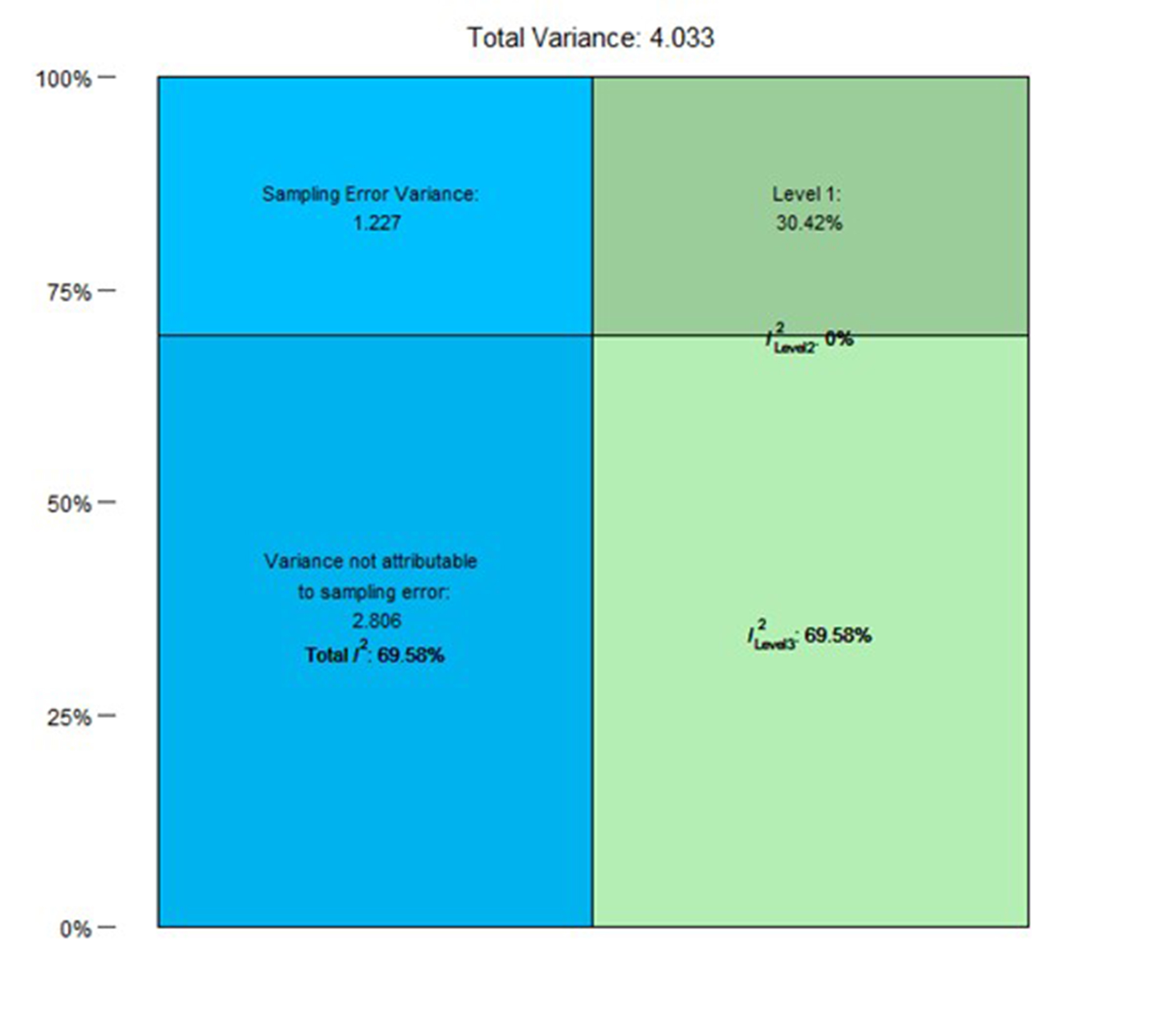

Supplement: Supplementary Figure 5 — The variance components identified by multilevel random effects in the milk production meta-analysis. Level 1 = sampling variance of the extracted effect size, level 2 = variance between effects sizes extracted from the same study, level 3 = variance among the studies selected for meta-analysis. [file Image_5.JPEG]

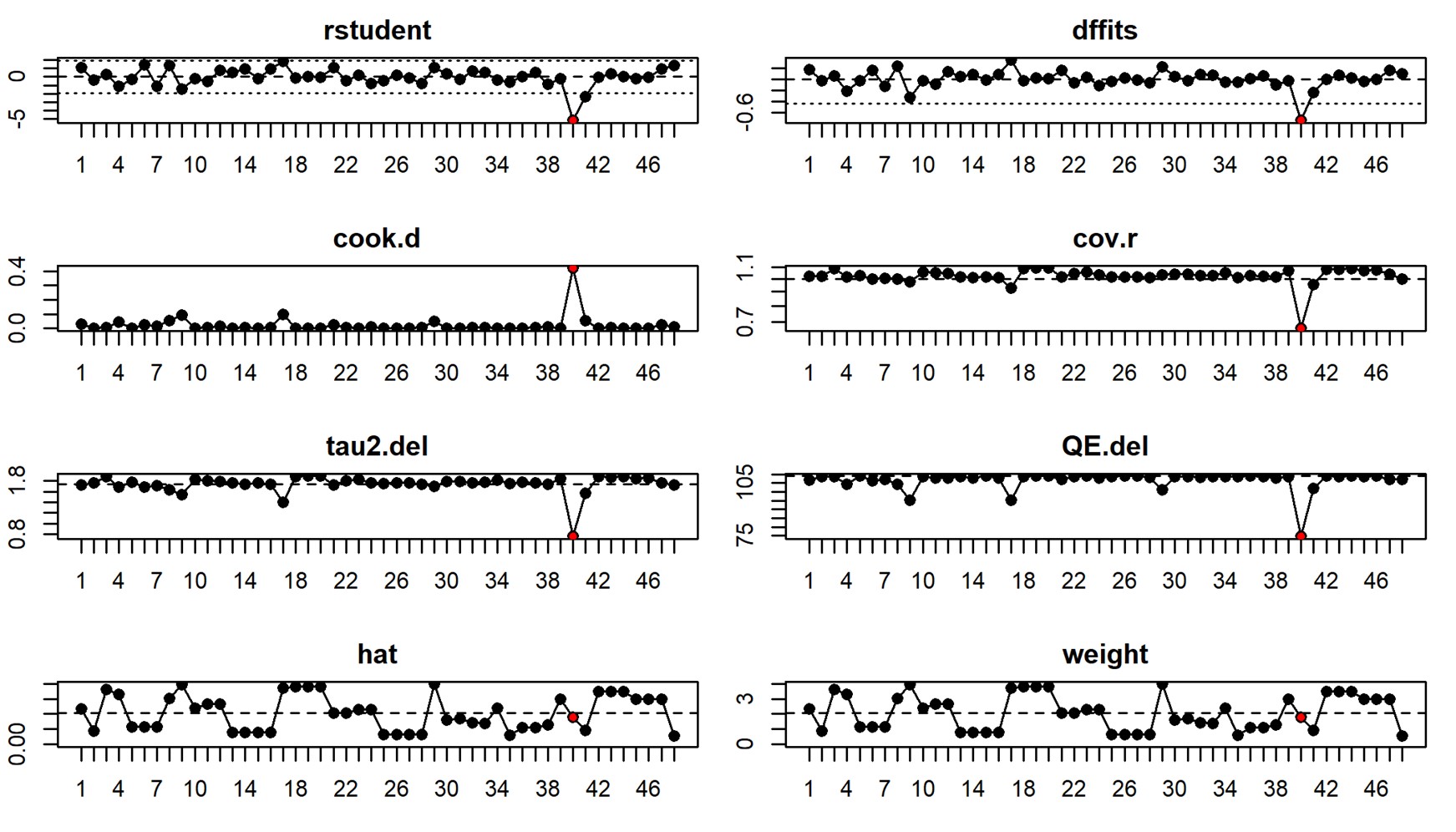

Supplement: Supplementary Figure 6 — Graphical presentation of the influence analysis of the milk production meta-analysis. [file Image_6.JPEG]

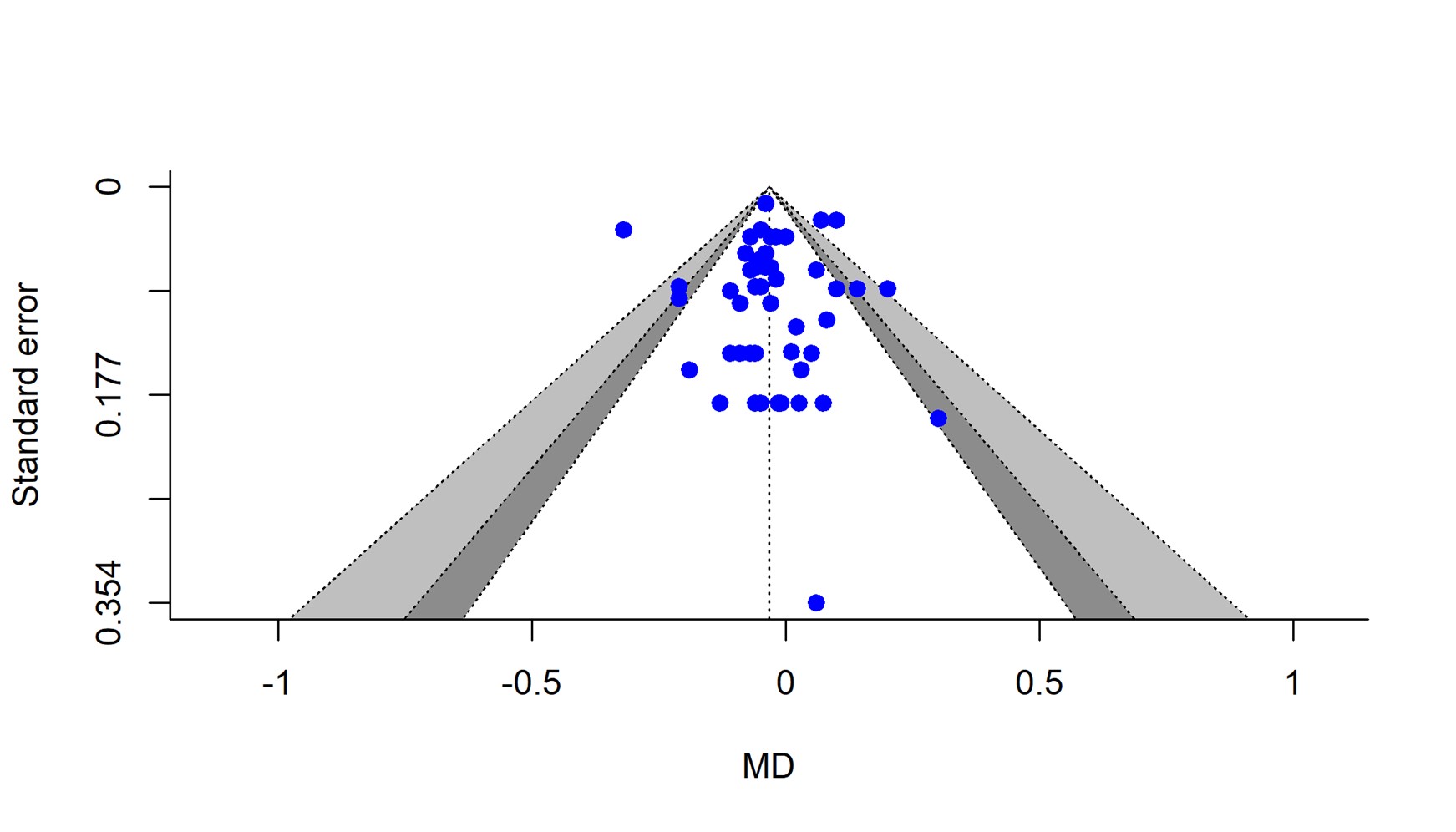

Supplement: Supplementary Figure 7 — A contour-enhanced funnel plot. The symmetrical distribution of studies around the mean difference (MD; x-axis) indicates that there were no publication biases in the studies included in the meta-analysis for milk protein content. The Egger's test was also not significant (P > 0.05). The vertical dotted line indicates the weighed MD effect size for milk protein content in chromium-supplemented cows. [file Image_7.JPEG]

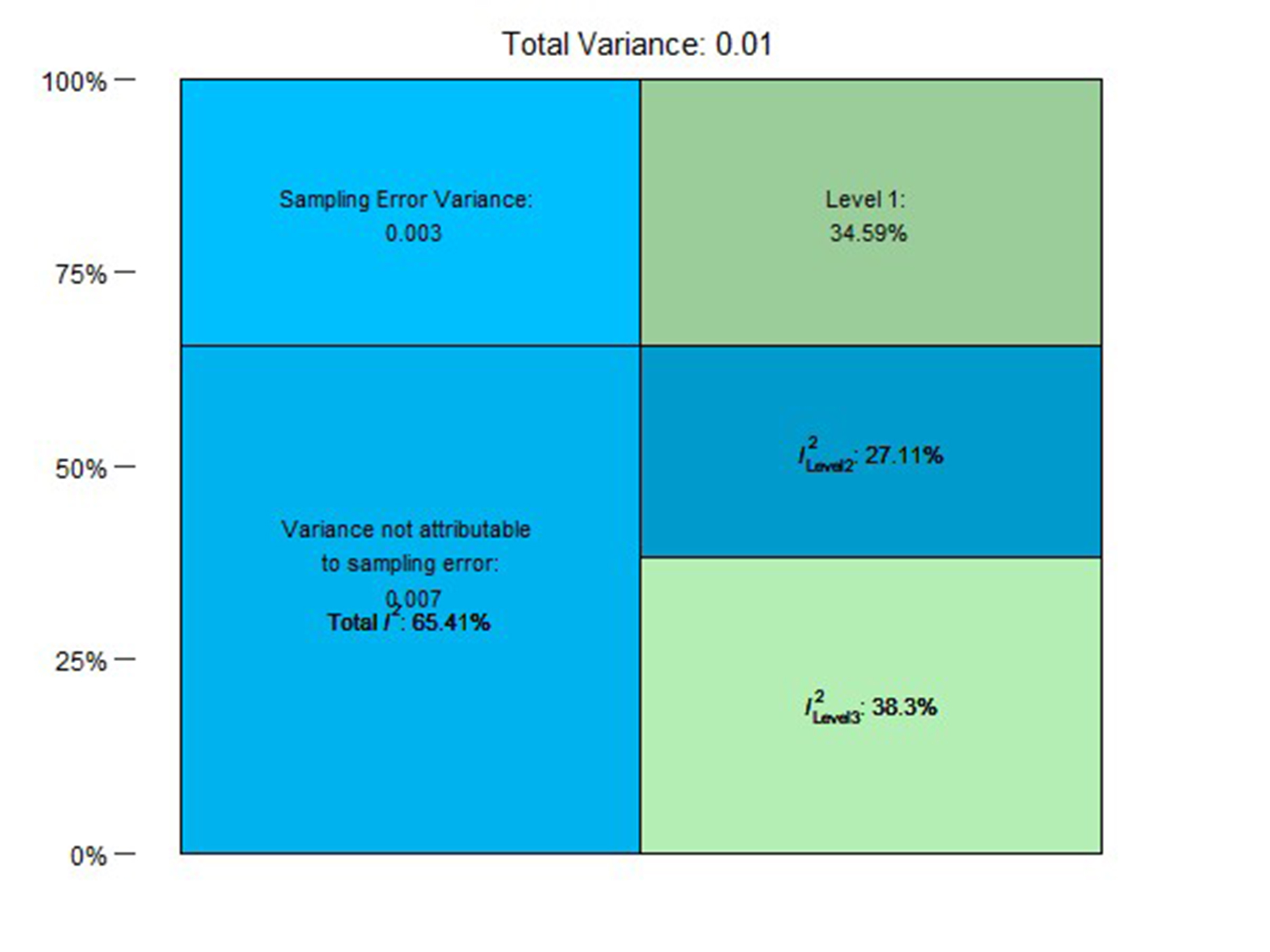

Supplement: Supplementary Figure 8 — The variance components identified by multilevel random effects in the milk protein content meta-analysis. Level 1 = sampling variance of the extracted effect size, level 2 = variance between effect sizes extracted from the same study, level 3 = variance among the studies selected for meta-analysis. [file Image_8.JPEG]

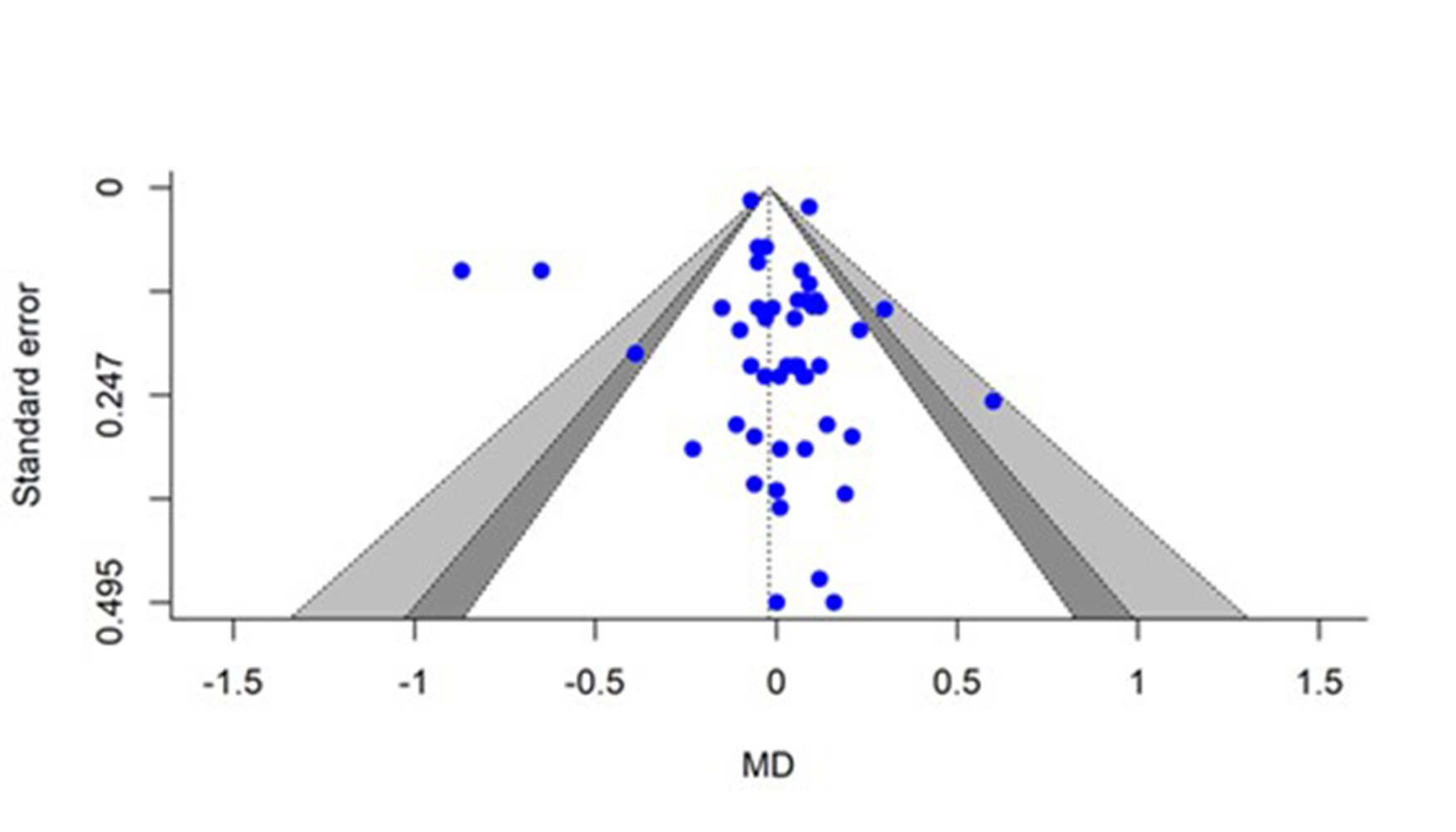

Supplement: Supplementary Figure 9 — A contour-enhanced funnel plot. The symmetrical distribution of studies around the mean difference (MD; x-axis) indicates that there were no publication biases in the studies included in the meta-analysis for milk fat content. The Egger's test was also not significant (P > 0.05). The vertical dotted line indicates the weighed MD effect size for milk fat content in chromium-supplemented cows. [file Image_9.JPEG]

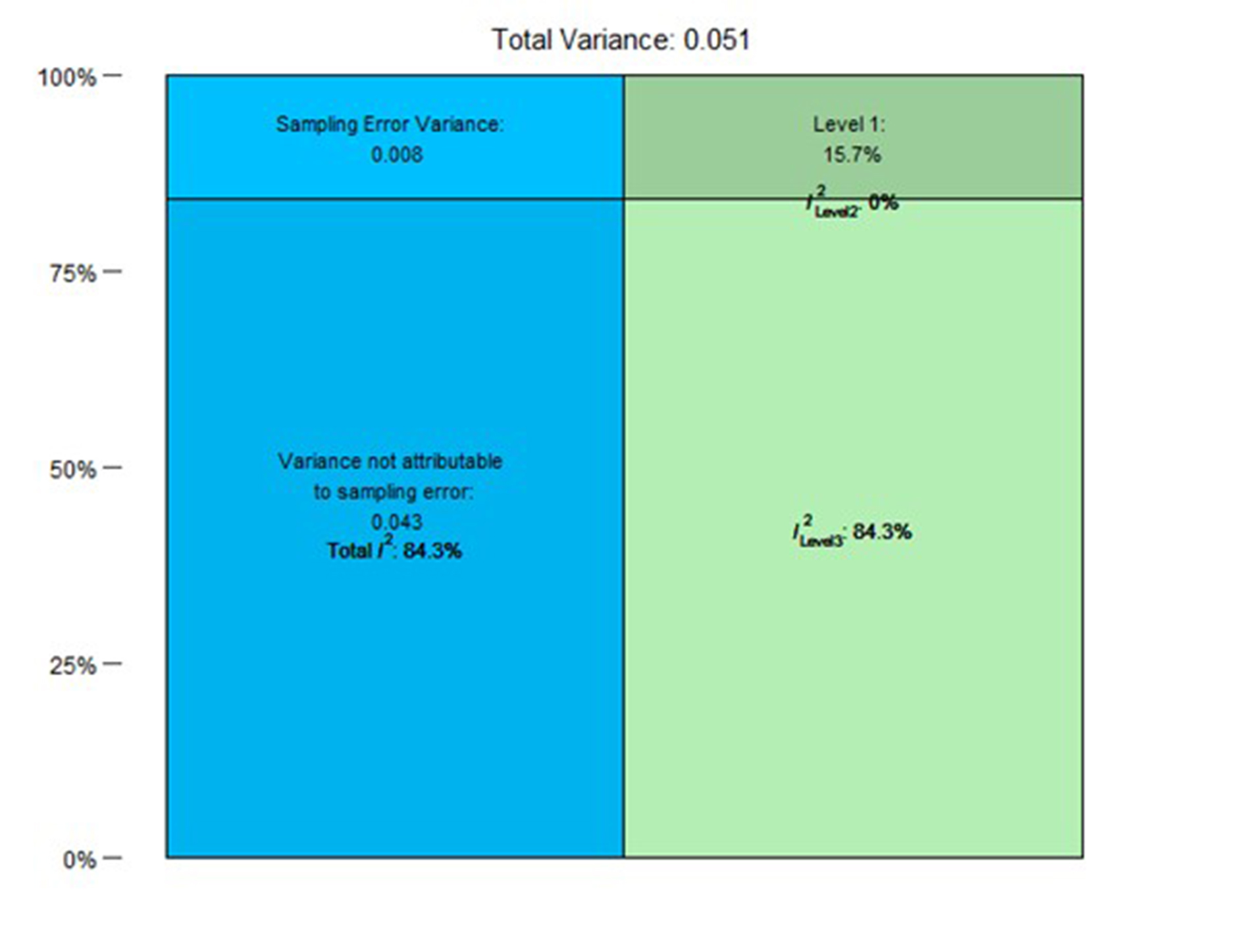

Supplement: Supplementary Figure 10 — The variance components identified by multilevel random effects in the milk fat content meta-analysis. Level 1 = sampling variance of the extracted effect size, level 2 = variance between effects sizes extracted from the same study, level 3 = variance among the studies selected for meta-analysis. [file Image_10.JPEG]

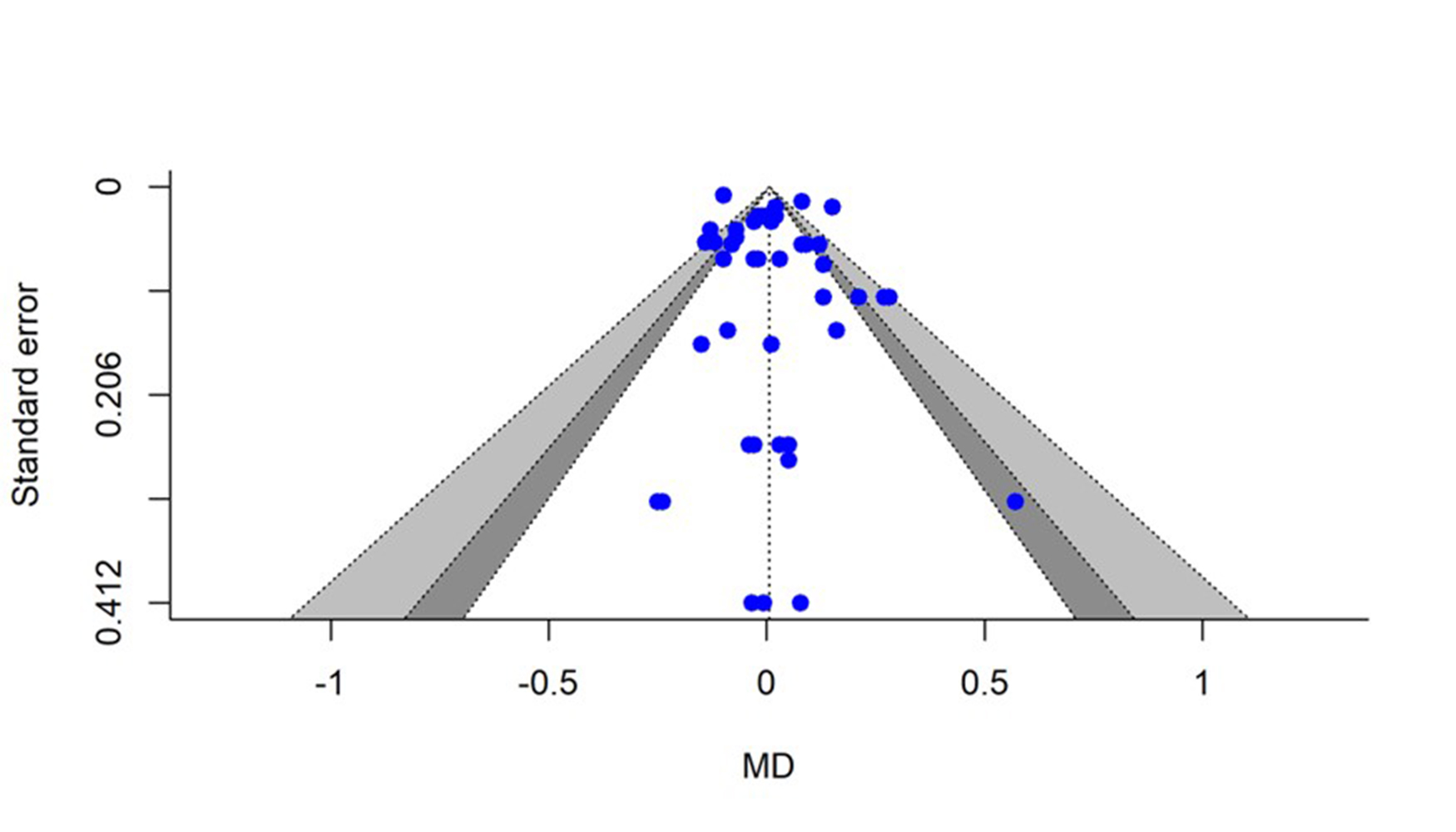

Supplement: Supplementary Figure 11 — A contour-enhanced funnel plot. The symmetrical distribution of studies around the mean difference (MD; x-axis) indicates that there were no publication biases in the studies included in the meta-analysis for milk lactose content. The Egger's test was also not significant (P > 0.05). The vertical dotted line indicates the weighed MD effect size for milk lactose content in chromium-supplemented cows. [file Image_11.JPEG]

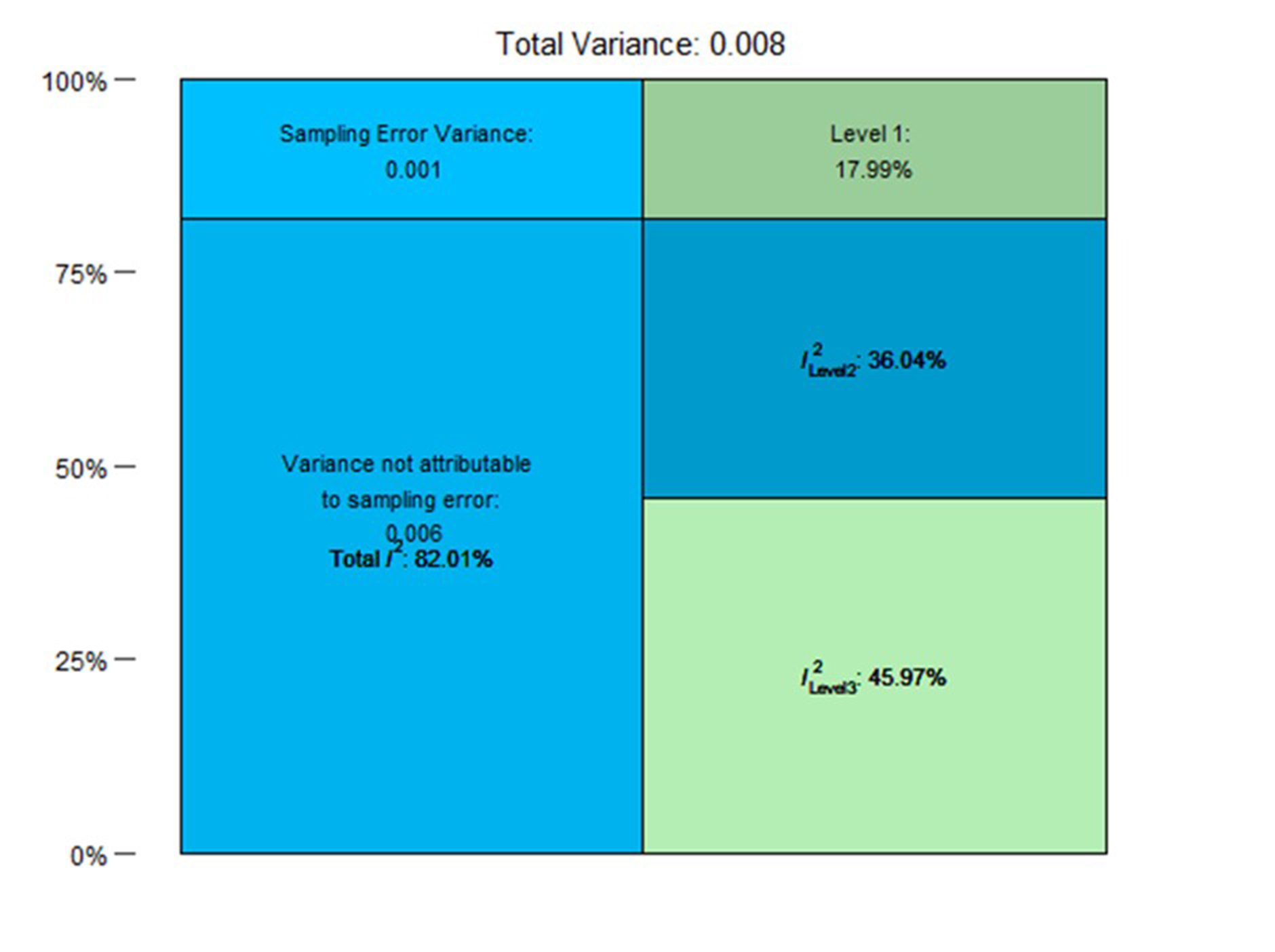

Supplement: Supplementary Figure 12 — The variance components identified by multilevel random effects in the milk lactose content meta-analysis. Level 1 = sampling variance of the extracted effect size, level 2 = variance between effects sizes extracted from the same study, level 3 = variance among the studies selected for meta-analysis. [file Image_12.JPEG]

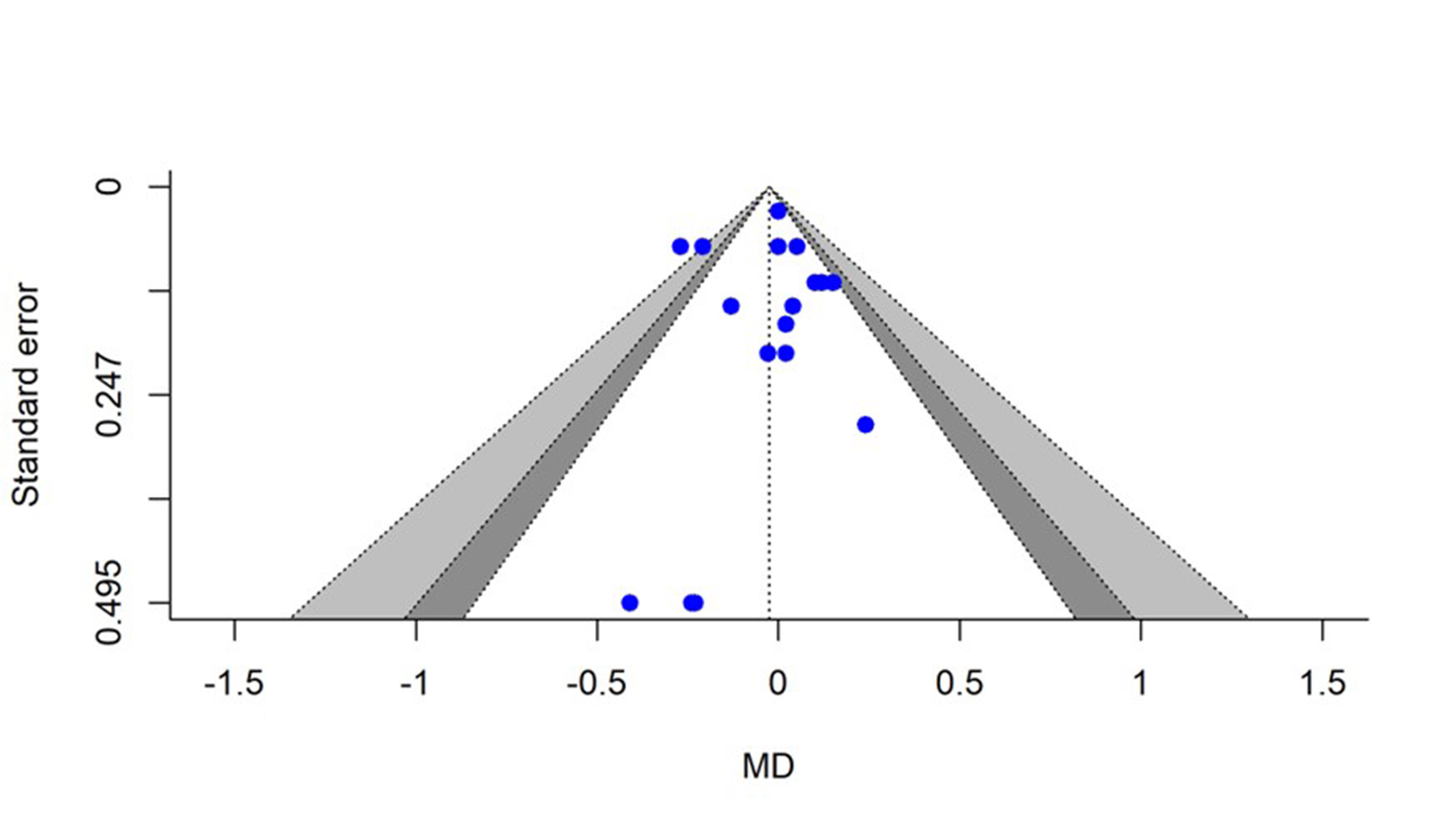

Supplement: Supplementary Figure 13 — A contour-enhanced funnel plot. The symmetrical distribution of studies around the mean difference (MD; x-axis) indicates that there were no publication biases in the studies included in the meta-analysis for solid-not-fat content. The Egger's test was also not significant (P > 0.05). The vertical dotted line indicates the weighed MD effect size for solid-not-fat content in chromium-supplemented cows. [file Image_13.JPEG]

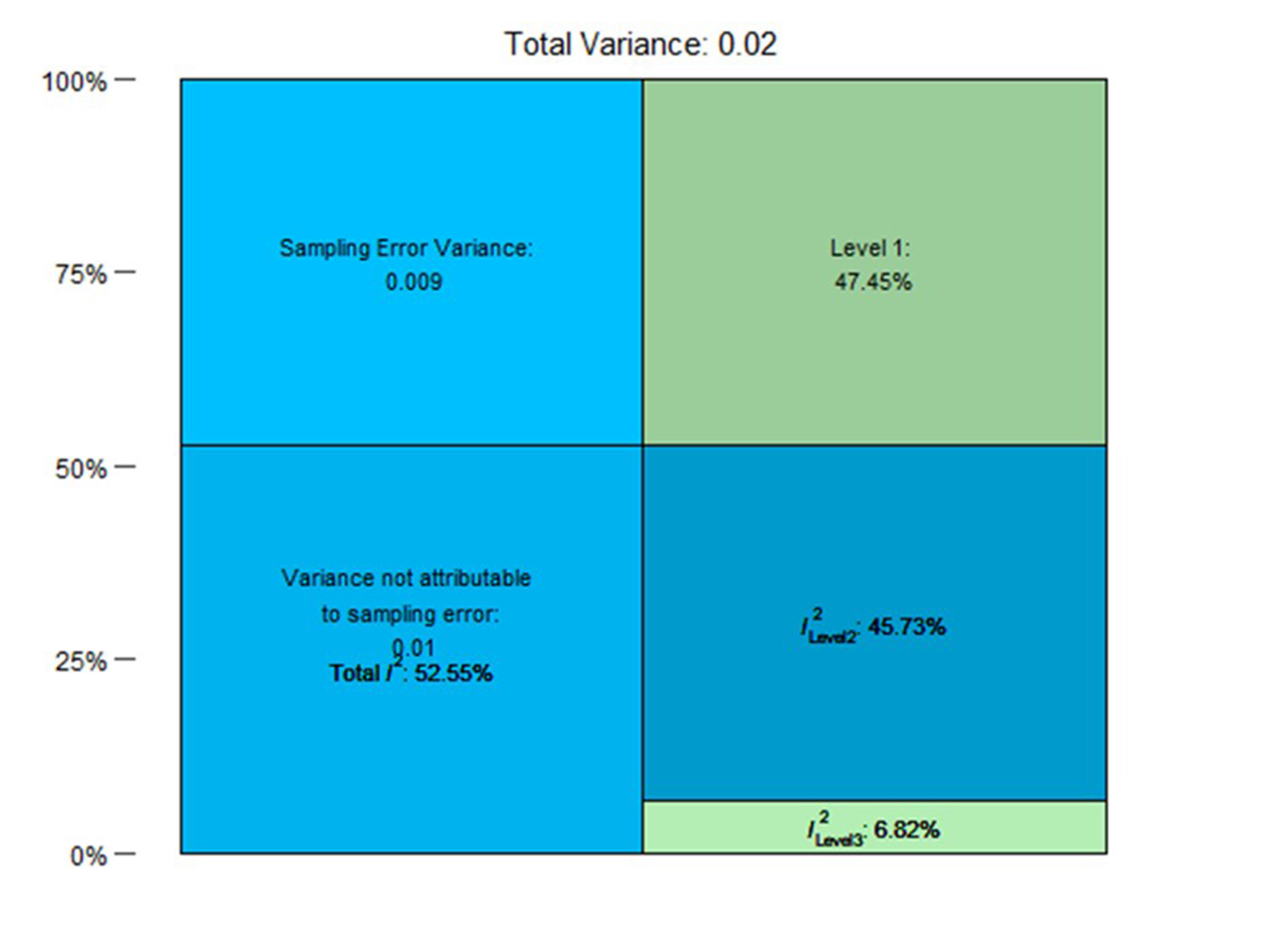

Supplement: Supplementary Figure 14 — The variance components identified by multilevel random effects in the solid-not-fat content meta-analysis. Level 1 = sampling variance of the extracted effect size, level 2 = variance between effects sizes extracted from the same study, level 3 = variance among the studies selected for meta-analysis. [file Image_14.JPEG]
