# Supplementary material for: Effects of dietary chromium supplementation on dry matter intake and milk production and composition in lactating dairy cows: A meta-analysis
Source: Front Vet Sci. 2023 Mar 16;10:1076777. doi: 10.3389/fvets.2023.1076777 (PMC10062059; doi:10.3389/fvets.2023.1076777)
Supplement: Supplementary file 15 [file Table_1.DOCX]

**Supplementary Table 1.** Summary of the studies and selected moderators for dry matter intake in dairy cows supplemented with chromium.

| Study | Cr supp^1^ | | | Control^2^ | | | Cr dose^3^ | BW^4^ | Exp. duration, wk^5^ | Parity^6^ | Parturition stage^7^ | Cr-complex^8^ |
| --- | --- | --- | --- | --- | --- | --- | --- | --- | --- | --- | --- | --- |
|  | N | M | SD | N | M | SD |  |  |  |  |  |  |
| Yang et al. (1996) | 6 | 16.8 | 2.20 | 6 | 16.4 | 2.20 | 5.50 | 532 | 22 | PP | AFP | Amino Acid |
| Yang et al. (1996) | 11 | 22.5 | 2.32 | 11 | 22.2 | 2.32 | 5.50 | 672 | 22 | MP | AFP | Amino Acid |
| Yang et al. (1996) | 9 | 15.5 | 1.20 | 9 | 15.1 | 1.20 | 7.75 | 643 | 22 | PP | BFP | Amino Acid |
| Yang et al. (1996) | 11 | 20.7 | 1.33 | 11 | 20.8 | 1.33 | 10.25 | 716 | 22 | MP | BFP | Amino Acid |
| Hayirli et al. (2001) | 10 | 11.1 | 1.26 | 10 | 10.9 | 1.26 | 3.90 | 720 | 7 | MP | BFP | Methionine |
| Hayirli et al. (2001) | 10 | 11.8 | 1.26 | 10 | 10.9 | 1.26 | 8.30 | 742 | 7 | MP | BFP | Methionine |
| Hayirli et al. (2001) | 11 | 12.5 | 1.33 | 10 | 10.9 | 1.26 | 16.50 | 739 | 7 | MP | BFP | Methionine |
| Hayirli et al. (2001) | 10 | 14.9 | 1.90 | 10 | 13.8 | 1.90 | 3.70 | 621 | 7 | MP | AFP | Methionine |
| Hayirli et al. (2001) | 10 | 17.2 | 1.90 | 10 | 13.8 | 1.90 | 7.70 | 636 | 7 | MP | AFP | Methionine |
| Hayirli et al. (2001) | 11 | 16.3 | 1.99 | 10 | 13.8 | 1.90 | 15.70 | 642 | 7 | MP | AFP | Methionine |
| Al-Saiady et al. (2004) | 80 | 21.24 | 2.50 | 80 | 19.56 | 2.68 | 5.00 | 600 | 10 | MP | AFP | Yeast |
| McNamara and Valdez (2005) | 10 | 11.4 | 1.42 | 10 | 10.6 | 1.42 | 10.00 |  | 8 | MP | BFP | Propionate |
| McNamara and Valdez (2005) | 10 | 23.1 | 2.21 | 10 | 20 | 2.21 | 10.00 |  | 8 | MP | AFP | Propionate |
| Smith et al. (2005) | 25 | 13.9 | 1.00 | 22 | 13.6 | 0.94 | 4.18 | 723 | 7 | MP | BFP | Methionine |
| Smith et al. (2005) | 25 | 13.6 | 1.00 | 22 | 13.6 | 0.94 | 8.37 | 723 | 7 | MP | BFP | Methionine |
| Smith et al. (2005) | 25 | 18.9 | 2.00 | 22 | 18.2 | 1.88 | 3.73 | 619 | 7 | MP | AFP | Methionine |
| Smith et al. (2005) | 25 | 19.7 | 2.00 | 22 | 18.2 | 1.88 | 7.62 | 619 | 7 | MP | AFP | Methionine |
| Soltan (2010) | 60 | 21.98 | 3.33 | 60 | 19.9 | 3.33 | 6.00 | 627 | 15 | MP+PP | AFP | Organic |
| An-Qiang et al. (2009) | 6 | 17.96 | 0.34 | 6 | 17.64 | 0.34 | 3.60 | 593 | 9 | MP | AFP | Picolinate |
| An-Qiang et al. (2009) | 6 | 18.18 | 0.34 | 6 | 17.64 | 0.34 | 7.20 | 593 | 9 | MP | AFP | Picolinate |
| An-Qiang et al. 2009 | 6 | 18.17 | 0.34 | 6 | 17.64 | 0.34 | 10.8 | 593 | 9 | MP | AFP | Picolinate |
| Sadri et al. (2009) | 8 | 12.7 | 0.99 | 8 | 11.6 | 0.99 | 10.94 | 705 | 7 | MP | BFP | Methionine |
| Sadri et al. (2009) | 8 | 11.9 | 0.99 | 8 | 12.1 | 0.99 | 10.88 | 700 | 7 | MP | BFP | Methionine |
| Sadri et al. (2009) | 8 | 18.4 | 1.67 | 8 | 16.9 | 1.67 | 10.32 | 652 | 7 | MP | AFP | Methionine |
| Sadri et al. (2009) | 8 | 17.8 | 1.67 | 8 | 18.3 | 1.67 | 10.17 | 633 | 7 | MP | AFP | Methionine |
| Mirzaei et al. (2011) | 5 | 24.2 | 1.19 | 5 | 21.8 | 1.19 | 6.21 | 620 | 7 | MP+PP | AFP | Methionine |
| Mirzaei et al. (2011) | 5 | 23.7 | 1.19 | 5 | 21.8 | 1.19 | 12.42 | 620 | 7 | MP+PP | AFP | Methionine |
| Jin et al. (2012) | 20 | 15.53 | 11.67 | 20 | 15.42 | 11.67 | 1.55 |  | 13 | PP | BFP | Propionate |
| Jin et al. (2012) | 20 | 16.36 | 11.67 | 20 | 15.42 | 11.67 | 3.27 |  | 13 | PP | BFP | Propionate |
| Jin et al. (2012) | 20 | 16.52 | 11.67 | 20 | 15.42 | 11.67 | 6.60 |  | 13 | PP | BFP | Propionate |
| Jin et al. (2012) | 20 | 16.43 | 11.67 | 20 | 15.42 | 11.67 | 9.85 |  | 13 | PP | BFP | Propionate |
| Vargas-Rodriguez et al. (2014) | 30 | 22.2 | 6.02 | 12 | 19.9 | 3.81 | 8.00 |  | 5 | MP+PP | AFP | Propionate |
| Yasui et al. (2014) | 30 | 16.5 | 1.64 | 31 | 15.8 | 1.67 | 8.00 | 714 | 12 | MP | BFP | Propionate |
| Yasui et al. (2014) | 30 | 22.9 | 1.64 | 31 | 22.9 | 1.67 | 8.00 | 6199 | 12 | MP | AFP | Propionate |
| Zhang et al. (2014) | 12 | 21.48 | 2.08 | 12 | 22.02 | 2.31 | 3.25 | 600 | 3 | PP | AFP | Picolinate |
| Zhang et al. (2014) | 12 | 23.7 | 4.21 | 12 | 20.89 | 2.29 | 3.25 | 600 | 3 | PP | AFP | Picolinate |
| Zhang et al. (2014) | 12 | 21.33 | 3.27 | 12 | 22.03 | 3.78 | 3.25 | 600 | 3 | PP | AFP | Picolinate |
| Rockwell and Allen (2016) | 24 | 11.8 | 2.06 | 24 | 12.7 | 2.06 | 8.00 | 780 | 8 | MP | BFP | Propionate |
| Rockwell and Allen (2016) | 12 | 18.5 | 2.32 | 12 | 18.1 | 2.32 | 8.00 | 780 | 8 | MP | AFP | Propionate |
| Rockwell and Allen (2016) | 12 | 18.8 | 2.32 | 12 | 18.6 | 2.32 | 8.00 | 780 | 8 | MP | AFP | Propionate |
| Pantelić et al. (2018) | 10 | 12.1 | 0.98 | 10 | 10.9 | 1.11 | 10.00 |  | 8 | MP | BFP | Yeast |
| Pantelić et al. (2018) | 10 | 15.9 | 4.24 | 10 | 16.7 | 2.75 | 10.00 |  | 8 | MP | AFP | Yeast |
| Pantelić et al. (2018) | 10 | 19.9 | 2.97 | 10 | 19 | 3.54 | 10.00 |  | 8 | MP | AFP | Yeast |
| Shan et al. (2020) | 6 | 18.1 | 0.71 | 6 | 17.2 | 0.71 | 3.25 |  | 10 | MP | AFP | Yeast |
| Shan et al. (2020) | 6 | 18.6 | 0.71 | 6 | 17.2 | 0.71 | 6.69 |  | 10 | MP | AFP | Yeast |
| Shan et al. (2020) | 6 | 18.1 | 0.71 | 6 | 17.2 | 0.71 | 9.77 |  | 10 | MP | AFP | Yeast |
| Wu et al. (2021) | 16 | 25.7 | 1.12 | 16 | 26.1 | 1.12 | 4.00 | 726 | 12 | MP | AFP | Methionine |
| Wu et al. (2021) | 16 | 26.3 | 1.12 | 16 | 26.1 | 1.12 | 8.00 | 726 | 12 | MP | AFP | Methionine |
| Wu et al. (2021) | 16 | 25.6 | 1.12 | 16 | 26.1 | 1.12 | 16.00 | 726 | 12 | MP | AFP | Methionine |

^1^Cr supp = chromium supplementation, N = the number of cows, M = mean, SD = standard deviation; ^2^Control, N = the number of cows, M = mean, SD = standard deviation; ^3^Cr dose = chromium supplementation/day/cow (mg); ^4^BW = body weight of the cows selected for meta-analysis; ^5^Exp. duration, wk. = experiment duration/duration of supplementation of chromium; ^6^Parity = parity of the cows (PP = primiparous, MP = multiparous, MP+PP = both primiparous and multiparous); ^7^Parturition stage, AFP = after parturition, BFP = before parturition; ^8^Cr-complex = complexes of chromium with other molecules like methionine, picolinate, and propionate.

**References:**

Al-Saiady, M., M. Al-Shaikh, S. Al-Mufarrej, T. Al-Showeimi, H. Mogawer, and A. Dirrar. 2004. Effect of chelated chromium supplementation on lactation performance and blood parameters of Holstein cows under heat stress. Anim. Feed Sci. Technol. 117(3-4):223-233.

An-Qiang, L., W. Zhi-Sheng, and Z. An-Guo. 2009. Effect of chromium picolinate supplementation on early lactation performance, rectal temperatures, respiration rates and plasma biochemical response of Holstein cows under heat stress. Pak. J. Nutr 8(7):940-945.

Hayirli, A., D. Bremmer, S. Bertics, M. Socha, and R. Grummer. 2001. Effect of chromium supplementation on production and metabolic parameters in periparturient dairy cows. J. Dairy Sci. 84(5):1218-1230.

Jin, X., S. Li, and W. Zhang. 2012. Effect of chromium propionate supplementation on lactation performance and blood parameters of dairy cows. J Anim Vet Adv 11(16):3031-3035.

McNamara, J. and F. Valdez. 2005. Adipose tissue metabolism and production responses to calcium propionate and chromium propionate. J. Dairy Sci. 88(7):2498-2507.

Mirzaei, M., G. Ghorbani, M. Khorvash, H. Rahmani, and A. Nikkhah. 2011. Chromium improves production and alters metabolism of early lactation cows in summer. J. Anim. Physiol. Anim. Nutr. 95(1):81-89.

Pantelić, M., L. J. Jovanović, R. Prodanović, I. Vujanac, M. Đurić, T. Ćulafić, S. Vranješ‐Đurić, G. Korićanac, and D. Kirovski. 2018. The impact of the chromium supplementation on insulin signalling pathway in different tissues and milk yield in dairy cows. J. Anim. Physiol. Anim. Nutr. 102(1):41-55.

Rockwell, R. and M. Allen. 2016. Chromium propionate supplementation during the peripartum period interacts with starch source fed postpartum: Production responses during the immediate postpartum and carryover periods. J. Dairy Sci. 99(6):4453-4463.

Sadri, H., G. Ghorbani, H. Rahmani, A. Samie, M. Khorvash, and R. Bruckmaier. 2009. Chromium supplementation and substitution of barley grain with corn: Effects on performance and lactation in periparturient dairy cows. J. Dairy Sci. 92(11):5411-5418.

Shan, Q., F. Ma, Y. Jin, D. Gao, H. Li, and P. Sun. 2020. Chromium yeast alleviates heat stress by improving antioxidant and immune function in Holstein mid-lactation dairy cows. Anim. Feed Sci. Technol. 269:114635.

Smith, K., M. Waldron, J. Drackley, M. Socha, and T. Overton. 2005. Performance of dairy cows as affected by prepartum dietary carbohydrate source and supplementation with chromium throughout the transition period. J. Dairy Sci. 88(1):255-263.

Soltan, M. 2010. Effect of dietary chromium supplementation on productive and reproductive performance of early lactating dairy cows under heat stress. J. Anim. Physiol. Anim. Nutr. 94(2):264-272.

Vargas-Rodriguez, C., K. Yuan, E. Titgemeyer, L. Mamedova, K. Griswold, and B. Bradford. 2014. Effects of supplemental chromium propionate and rumen-protected amino acids on productivity, diet digestibility, and energy balance of peak-lactation dairy cattle. J. Dairy Sci. 97(6):3815-3821.

Wu, Z., W. Peng, J. Liu, G. Xu, and D. Wang. 2021. Effect of chromium methionine supplementation on lactation performance, hepatic respiratory rate and anti-oxidative capacity in early-lactating dairy cows. Animal 15(9):100326.

Yang, W., D. Mowat, A. Subiyatno, and R. Liptrap. 1996. Effects of chromium supplementation on early lactation performance of Holstein cows. Canadian Journal of Animal Science 76(2):221-230.

Yasui, T., J. McArt, C. Ryan, R. Gilbert, D. Nydam, F. Valdez, K. Griswold, and T. Overton. 2014. Effects of chromium propionate supplementation during the periparturient period and early lactation on metabolism, performance, and cytological endometritis in dairy cows. J. Dairy Sci. 97(10):6400-6410.

Zhang, F., X. Weng, J. Wang, D. Zhou, W. Zhang, C. Zhai, Y. Hou, and Y. Zhu. 2014. Effects of temperature–humidity index and chromium supplementation on antioxidant capacity, heat shock protein 72, and cytokine responses of lactating cows. J. Anim. Sci. 92(7):3026-3034.
