# Supplementary material for: Effects of dietary chromium supplementation on dry matter intake and milk production and composition in lactating dairy cows: A meta-analysis
Source: Front Vet Sci. 2023 Mar 16;10:1076777. doi: 10.3389/fvets.2023.1076777 (PMC10062059; doi:10.3389/fvets.2023.1076777)
Supplement: Supplementary file 16 [file Table_2.DOCX]

**Supplementary Table 2 |** Summary of the studies and selected moderators for milk production meta-analysis in dairy cows supplemented with chromium.

| Study | Cr supp^1^ | | | Control^2^ | | | Cr dose^3^ | BW^4^ | Exp. duration, wk^5^ | Parity^6^ | DIM^7^ | Cr-complex^8^ |
| --- | --- | --- | --- | --- | --- | --- | --- | --- | --- | --- | --- | --- |
|  | N | M | SD | N | M | SD |  |  |  |  |  |  |
| Yang et al. (1996) | 6 | 27.50 | 2.94 | 6 | 24.30 | 1.20 | 5.50 | 532 | 22 | PP | 1 | Amino Acid |
| Yang et al. (1996) | 11 | 36.60 | 6.30 | 11 | 36.40 | 6.30 | 5.50 | 672 | 22 | MP | 1 | Amino Acid |
| Yang et al. (1996) | 9 | 25.70 | 1.50 | 9 | 24.10 | 1.50 | 7.75 | 643 | 22 | PP | 1 | Amino Acid |
| Yang et al. (1996) | 11 | 36.30 | 1.99 | 11 | 36.80 | 1.99 | 10.25 | 716 | 22 | MP | 1 | Amino Acid |
| Hayirli et al. (2001) | 10 | 34.00 | 5.06 | 10 | 33.50 | 5.06 | 3.70 | 621 | 7 | MP | 1 | Methionine |
| Hayirli et al. (2001) | 10 | 38.50 | 5.06 | 10 | 33.50 | 5.06 | 7.70 | 636 | 7 | MP | 1 | Methionine |
| Hayirli et al. (2001) | 11 | 31.80 | 5.31 | 10 | 33.50 | 5.06 | 15.70 | 642 | 7 | MP | 1 | Methionine |
| Al-Saiady et al. (2004) | 80 | 33.24 | 5.19 | 80 | 29.87 | 6.80 | 5.00 | 600 | 10 | MP | 1 | Yeast |
| Bryan et al. (2004) | 110 | 26.00 | 4.20 | 122 | 26.70 | 4.42 | 6.25 | 514 | 27 | MP+PP | 1 | Methionine |
| McNamara and Valdez (2005) | 10 | 41.60 | 2.88 | 10 | 40.80 | 2.88 | 10.00 |  | 8 | MP | 1 | Propionate |
| Smith et al. (2005) | 25 | 40.50 | 4.00 | 22 | 40.30 | 3.75 | 3.73 | 619 | 7 | MP | 1 | Methionine |
| Smith et al. (2005) | 25 | 42.80 | 4.00 | 22 | 40.30 | 3.75 | 7.62 | 619 | 7 | MP | 1 | Methionine |
| Terramoccia et al. (2005) | 4 | 30.94 | 4.18 | 4 | 28.10 | 4.18 | 11.28 | 531 | 10 | PP | 1 | Amino Acid |
| Terramoccia et al. (2005) | 4 | 32.45 | 4.18 | 4 | 28.10 | 4.18 | 22.56 | 531 | 10 | PP | 1 | Amino Acid |
| Terramoccia et al. (2005) | 3 | 34.53 | 3.62 | 3 | 33.96 | 3.62 | 11.28 | 605 | 10 | MP | 1 | Amino Acid |
| Terramoccia et al. (2005) | 3 | 38.33 | 3.62 | 3 | 33.96 | 3.62 | 22.56 | 605 | 10 | MP | 1 | Amino Acid |
| Soltan (2010) | 60 | 34.90 | 3.64 | 60 | 31.20 | 3.61 | 6.00 | 627 | 15 | MP+PP | 1 | Organic |
| An-Qiang et al. (2009) | 6 | 25.29 | 1.05 | 6 | 24.34 | 1.05 | 3.6.00 | 593 | 9 | MP | 21 | Picolinate |
| An-Qiang et al. (2009 | 6 | 25.63 | 1.05 | 6 | 24.34 | 1.05 | 7.20 | 593 | 9 | MP | 21 | Picolinate |
| An-Qiang et al. (2009 | 6 | 25.52 | 1.05 | 6 | 24.34 | 1.05 | 10.80 | 593 | 9 | MP | 21 | Picolinate |
| Sadri et al. (2009) | 8 | 37.70 | 2.94 | 8 | 34.30 | 2.94 | 10.32 | 652 | 7 | MP | 1 | Methionine |
| Sadri et al. (2009) | 8 | 35.20 | 2.94 | 8 | 34.90 | 2.94 | 10.17 | 633 | 7 | MP | 1 | Methionine |
| Mirzaei et al. (2011) | 5 | 39.60 | 2.12 | 5 | 38.00 | 2.12 | 6.21 | 620 | 7 | MP+PP | 38 | Methionine |
| Mirzaei et al. (2011) | 5 | 37.80 | 2.12 | 5 | 38.00 | 2.12 | 12.42 | 620 | 7 | MP+PP | 38 | Methionine |
| Jin et al. (2012) | 20 | 25.05 | 10.29 | 20 | 25.39 | 10.29 | 1.55 |  | 13 | PP | 1 | Propionate |
| Jin et al. (2012) | 20 | 27.36 | 10.29 | 20 | 25.39 | 10.29 | 3.27 |  | 13 | PP | 1 | Propionate |
| Jin et al. (2012) | 20 | 26.06 | 10.29 | 20 | 25.39 | 10.29 | 6.60 |  | 13 | PP | 1 | Propionate |
| Jin et al. (2012) | 20 | 23.84 | 10.29 | 20 | 25.39 | 10.29 | 9.85 |  | 13 | PP | 1 | Propionate |
| Kafilzadeh and Targhibi (2012) | 30 | 38.37 | 2.08 | 30 | 35.62 | 2.08 | 8.00 | 682 | 6 | MP | 1 | Methionine |
| Pechova et al. (2003) | 9 | 26.60 | 3.60 | 9 | 24.60 | 4.00 | 5.00 |  | 7 | PP | 1 | Lactate |
| Pechova et al. (2003) | 15 | 32.50 | 4.20 | 15 | 31.90 | 5.30 | 5.00 |  | 7 | MP | 1 | Lactate |
| Targhibi et al. (2012) | 30 | 37.01 | 7.67 | 30 | 34.08 | 7.67 | 8.00 | 682 | 6 | MP | 1 | Methionine |
| Vargas-Rodriguez et al. (2014) | 12 | 43.07 | 4.99 | 12 | 40.50 | 4.99 | 8.00 |  | 5 | MP+PP | 38 | Propionate |
| Yasui et al. (2014) | 30 | 42.50 | 4.93 | 31 | 42.00 | 5.01 | 8.00 | 720 | 12 | MP | 1 | Propionate |
| Leiva et al. (2015) | 6 | 19.70 | 5.88 | 6 | 20.80 | 5.88 | 10.0 | 537 | 30 | MP+PP | 76 | Propionate |
| Rockwell and Allen (2016) | 12 | 39.90 | 5.72 | 12 | 38.50 | 5.72 | 8.00 | 780 | 8 | MP | 1 | Propionate |
| Rockwell and Allen (2016) | 12 | 44.10 | 5.72 | 12 | 41.40 | 5.72 | 8.00 | 780 | 8 | MP | 1 | Propionate |
| Leiva et al. (2017) | 14 | 21.50 | 5.61 | 12 | 22.50 | 5.20 | 10.00 | 574 | 26 | MP | 1 | Propionate |
| Leiva et al. (2018) | 20 | 20.50 | 3.13 | 20 | 19.70 | 3.13 | 10.00 | 590 | 29 | MP | 81 | Propionate |
| Pantelić et al. (2018) | 10 | 17.20 | 0.63 | 10 | 25.60 | 5.22 | 10.00 |  | 8 | MP | 1 | Yeast |
| Pantelić et al. (2018) | 10 | 23.60 | 5.09 | 10 | 29.30 | 6.64 | 10.00 |  | 8 | MP | 1 | Yeast |
| Shan et al. (2020) | 6 | 23.60 | 1.32 | 6 | 22.50 | 1.32 | 3.25 |  | 10 | MP | 105 | Yeast |
| Shan et al. (2020) | 6 | 24.20 | 1.32 | 6 | 22.50 | 1.32 | 6.69 |  | 10 | MP | 105 | Yeast |
| Shan et al. (2020) | 6 | 23.80 | 1.32 | 6 | 22.50 | 1.32 | 9.77 |  | 10 | MP | 105 | Yeast |
| Wu et al. (2021) | 16 | 33.20 | 2.76 | 16 | 32.40 | 2.76 | 4.00 | 726 | 12 | MP | 50 | Methionine |
| Wu et al. (2021) | 16 | 33.50 | 2.76 | 16 | 32.40 | 2.76 | 8.00 | 726 | 12 | MP | 50 | Methionine |
| Wu et al. (2021) | 16 | 35.10 | 2.76 | 16 | 32.40 | 2.76 | 16.00 | 726 | 12 | MP | 50 | Methionine |
| Khalili et al. (2011) | 10 | 50.80 | 8.13 | 10 | 44.25 | 8.13 | 5.00 |  | 17 | MP | 1 | Methionine |

^1^Cr supp = chromium supplementation, N = the number of cows, M = mean, SD = standard deviation; ^2^Control, N = the number of cows, M = mean, SD = standard deviation; ^3^Cr dose = chromium supplemented (mg)/day/cow; ^4^BW = body weight of the cows selected for meta-analysis; ^5^Exp. duration, wk. = experiment duration/duration of supplementation of chromium; ^6^Parity = parity of the cows (PP = primiparous, MP = multiparous, MP+PP = both primiparous and multiparous); ^7^DIM = days in milk; ^8^Cr-complex = complexes of chromium with other molecules like methionine, picolinate, and propionate.

**References:**

Al-Saiady, M., M. Al-Shaikh, S. Al-Mufarrej, T. Al-Showeimi, H. Mogawer, and A. Dirrar. 2004. Effect of chelated chromium supplementation on lactation performance and blood parameters of Holstein cows under heat stress. Animal Feed Science and Technology 117(3-4):223-233.

An-Qiang, L., W. Zhi-Sheng, and Z. An-Guo. 2009. Effect of chromium picolinate supplementation on early lactation performance, rectal temperatures, respiration rates and plasma biochemical response of Holstein cows under heat stress. Pak. J. Nutr 8(7):940-945.

Bryan, M., M. Socha, and D. Tomlinson. 2004. Supplementing intensively grazed late-gestation and early-lactation dairy cattle with chromium. Journal of Dairy Science 87(12):4269-4277.

Hayirli, A., D. Bremmer, S. Bertics, M. Socha, and R. Grummer. 2001. Effect of chromium supplementation on production and metabolic parameters in periparturient dairy cows. Journal of Dairy Science 84(5):1218-1230.

Jin, X., S. Li, and W. Zhang. 2012. Effect of chromium propionate supplementation on lactation performance and blood parameters of dairy cows. J Anim Vet Adv 11(16):3031-3035.

Kafilzadeh, F. and M. Targhibi. 2012. Effect of chromium supplementation on productive and reproductive performances and some metabolic parameters in late gestation and early lactation of dairy cows. Biological Trace Element Research 149(1):42-49.

Khalili, M., A. Foroozandeh, and M. Toghyani. 2011. Lactation performance and serum biochemistry of dairy cows fed supplemental chromium in the transition period. African Journal of Biotechnology 10(50):10304-10310.

Leiva, T., R. Cooke, A. Brandão, A. Aboin, J. Ranches, and J. Vasconcelos. 2015. Effects of excessive energy intake and supplementation with chromium propionate on insulin resistance parameters, milk production, and reproductive outcomes of lactating dairy cows. Livestock Science 180:121-128.

Leiva, T., R. Cooke, A. Brandão, R. Bertin, E. Colombo, V. Miranda, L. Lourenço, S. Rodrigues, and J. Vasconcelos. 2018. Effects of supplemental calcium salts of palm oil and chromium-propionate on insulin sensitivity and productive and reproductive traits of mid-to late-lactating Holstein× Gir dairy cows consuming excessive energy. Journal of dairy science 101(1):491-504.

Leiva, T., R. Cooke, A. Brandão, U. Pardelli, R. Rodrigues, F. Corrá, and J. Vasconcelos. 2017. Effects of concentrate type and chromium propionate on insulin sensitivity, productive and reproductive parameters of lactating dairy cows consuming excessive energy. Animal 11(3):436-444.

McNamara, J. and F. Valdez. 2005. Adipose tissue metabolism and production responses to calcium propionate and chromium propionate. Journal of Dairy Science 88(7):2498-2507.

Mirzaei, M., G. Ghorbani, M. Khorvash, H. Rahmani, and A. Nikkhah. 2011. Chromium improves production and alters metabolism of early lactation cows in summer. Journal of animal physiology and animal nutrition 95(1):81-89.

Pantelić, M., L. J. Jovanović, R. Prodanović, I. Vujanac, M. Đurić, T. Ćulafić, S. Vranješ‐Đurić, G. Korićanac, and D. Kirovski. 2018. The impact of the chromium supplementation on insulin signalling pathway in different tissues and milk yield in dairy cows. Journal of animal physiology and animal nutrition 102(1):41-55.

Pechova, A., S. Cech, L. Pavlata, and A. Podhorsky. 2003. The influence of chromium supplementation on metabolism, performance and reproduction of dairy cows in a herd with increased occurrence of ketosis. Czech Journal of Animal Science-UZPI (Czech Republic).

Rockwell, R. and M. Allen. 2016. Chromium propionate supplementation during the peripartum period interacts with starch source fed postpartum: Production responses during the immediate postpartum and carryover periods. Journal of Dairy Science 99(6):4453-4463.

Sadri, H., G. Ghorbani, H. Rahmani, A. Samie, M. Khorvash, and R. Bruckmaier. 2009. Chromium supplementation and substitution of barley grain with corn: Effects on performance and lactation in periparturient dairy cows. Journal of Dairy Science 92(11):5411-5418.

Shan, Q., F. Ma, Y. Jin, D. Gao, H. Li, and P. Sun. 2020. Chromium yeast alleviates heat stress by improving antioxidant and immune function in Holstein mid-lactation dairy cows. Anim. Feed Sci. Technol. 269:114635.

Smith, K., M. Waldron, J. Drackley, M. Socha, and T. Overton. 2005. Performance of dairy cows as affected by prepartum dietary carbohydrate source and supplementation with chromium throughout the transition period. Journal of Dairy Science 88(1):255-263.

Soltan, M. 2010. Effect of dietary chromium supplementation on productive and reproductive performance of early lactating dairy cows under heat stress. Journal of animal physiology and animal nutrition 94(2):264-272.

Targhibi, M., H. K. Shabankareh, and F. Kafilzadeh. 2012. Effects of supplemental chromium on lactation and some blood parameters of dairy cows in late gestation and early lactation. Asian Journal of Animal and Veterinary Advances 7(11):1205-1211.

Terramoccia, S., S. Bartocci, and E. Lillini. 2005. Milk yield and immune response of periparturient and early lactation Friesian cows fed diets supplemented with a high level of amino-acid chelated chromium. Asian-australasian journal of animal sciences 18(8):1098-1104.

Vargas-Rodriguez, C., K. Yuan, E. Titgemeyer, L. Mamedova, K. Griswold, and B. Bradford. 2014. Effects of supplemental chromium propionate and rumen-protected amino acids on productivity, diet digestibility, and energy balance of peak-lactation dairy cattle. Journal of Dairy Science 97(6):3815-3821.

Wu, Z., W. Peng, J. Liu, G. Xu, and D. Wang. 2021. Effect of chromium methionine supplementation on lactation performance, hepatic respiratory rate and anti-oxidative capacity in early-lactating dairy cows. Animal 15(9):100326.

Yang, W., D. Mowat, A. Subiyatno, and R. Liptrap. 1996. Effects of chromium supplementation on early lactation performance of Holstein cows. Canadian Journal of Animal Science 76(2):221-230.

Yasui, T., J. McArt, C. Ryan, R. Gilbert, D. Nydam, F. Valdez, K. Griswold, and T. Overton. 2014. Effects of chromium propionate supplementation during the periparturient period and early lactation on metabolism, performance, and cytological endometritis in dairy cows. Journal of Dairy Science 97(10):6400-6410.
