# Supplementary material for: Effects of dietary chromium supplementation on dry matter intake and milk production and composition in lactating dairy cows: A meta-analysis
Source: Front Vet Sci. 2023 Mar 16;10:1076777. doi: 10.3389/fvets.2023.1076777 (PMC10062059; doi:10.3389/fvets.2023.1076777)
Supplement: Supplementary file 20 [file Table_6.DOCX]

**Supplementary Table 6.** Summary of the studies and selected moderators for solids-not-fat meta-analysis in dairy cows supplemented with chromium.

| Study | Cr supp^1^ | | | Control^2^ | | | Cr dose^3^ | BW^4^ | Exp. Duration, wk^5^ | Parity^6^ | DIM^7^ | Cr-complexes^8^ |
| --- | --- | --- | --- | --- | --- | --- | --- | --- | --- | --- | --- | --- |
|  | N | M | SD | N | M | SD |  |  |  |  |  |  |
| Yang et al. (1996) | 6 | 9.05 | 0.490 | 6 | 8.81 | 0.490 | 5.50 | 532 | 22 | PP | 1 | Amino Acid |
| Yang et al. (1996) | 11 | 8.74 | 0.332 | 11 | 8.87 | 0.332 | 5.50 | 672 | 22 | MP | 1 | Amino Acid |
| Yang et al. (1996) | 9 | 9.01 | 0.300 | 9 | 8.97 | 0.300 | 7.75 | 643 | 22 | PP | 1 | Amino Acid |
| Yang et al. (1996) | 11 | 8.74 | 0.166 | 11 | 8.95 | 0.166 | 10.25 | 716 | 22 | MP | 1 | Amino Acid |
| Hayirli et al. (2001) | 10 | 8.41 | 1.107 | 10 | 8.82 | 1.107 | 3.70 | 621 | 7 | MP | 1 | Methionine |
| Hayirli et al. (2001) | 10 | 8.59 | 1.107 | 10 | 8.82 | 1.107 | 7.70 | 636 | 7 | MP | 1 | Methionine |
| Hayirli et al. (2001) | 11 | 8.58 | 1.161 | 10 | 8.82 | 1.107 | 15.70 | 642 | 7 | MP | 1 | Methionine |
| Al-Saiady et al. (2004) | 30 | 8.2 | 0.274 | 30 | 8.20 | 0.274 | 5.00 | 600 | 10 | MP | 1 | Yeast |
| Smith et al. (2005) | 25 | 8.99 | 0.250 | 22 | 8.94 | 0.235 | 3.73 | 619 | 7 | MP | 1 | Methionine |
| Smith et al. (2005) | 25 | 8.67 | 0.250 | 22 | 8.94 | 0.235 | 7.62 | 619 | 7 | MP | 1 | Methionine |
| An-Qiang et al. (2009) | 6 | 8.06 | 0.343 | 6 | 8.04 | 0.343 | 3.60 | 593 | 9 | MP | 21 | Picolinate |
| An-Qiang et al. (2009) | 6 | 8.01 | 0.343 | 6 | 8.04 | 0.343 | 7.20 | 593 | 9 | MP | 21 | Picolinate |
| An-Qiang et al. (2009) | 6 | 8.01 | 0.343 | 6 | 8.04 | 0.343 | 10.80 | 593 | 9 | MP | 21 | Picolinate |
| Kafilzadeh and Targhibi (2012) | 30 | 8.59 | 0.110 | 30 | 8.59 | 0.110 | 8.00 | 682 | 6 | MP | 1 | Methionine |
| Shan et al. (2020) | 6 | 8.62 | 0.196 | 6 | 8.52 | 0.196 | 3.25 |  | 10 | MP | 105 | Yeast |
| Shan et al. (2020) | 6 | 8.64 | 0.196 | 6 | 8.52 | 0.196 | 6.70 |  | 10 | MP | 105 | Yeast |
| Shan et al. (2020) | 6 | 8.67 | 0.196 | 6 | 8.52 | 0.196 | 9.77 |  | 10 | MP | 105 | Yeast |
| Khalili et al. (2011) | 10 | 8.42 | 0.364 | 10 | 8.40 | 0.364 | 5.00 |  | 17 | MP | 1 | Methionine |

^1^Cr supp = chromium supplementation, N = the number of cows, M = mean, SD = standard deviation; ^2^Control, N = the number of cows, M = mean, SD = standard deviation; ^3^Cr dose = chromium supplemented (mg)/day/cow; ^4^BW = body weight of the cows selected for meta-analysis; ^5^Exp. duration, wk. = experiment duration/duration of supplementation of chromium; ^6^Parity = parity of the cows (PP = primiparous, MP = multiparous, MP+PP = both primiparous and multiparous); ^7^DIM = days in milk; ^8^Cr-complex = complexes of chromium with other molecules such as methionine, picolinate, and propionate.

**References:**

Al-Saiady, M., M. Al-Shaikh, S. Al-Mufarrej, T. Al-Showeimi, H. Mogawer, and A. Dirrar. 2004. Effect of chelated chromium supplementation on lactation performance and blood parameters of Holstein cows under heat stress. Animal Feed Science and Technology 117(3-4):223-233.

An-Qiang, L., W. Zhi-Sheng, and Z. An-Guo. 2009. Effect of chromium picolinate supplementation on early lactation performance, rectal temperatures, respiration rates and plasma biochemical response of Holstein cows under heat stress. Pak. J. Nutr 8(7):940-945.

Hayirli, A., D. Bremmer, S. Bertics, M. Socha, and R. Grummer. 2001. Effect of chromium supplementation on production and metabolic parameters in periparturient dairy cows. Journal of Dairy Science 84(5):1218-1230.

Kafilzadeh, F. and M. Targhibi. 2012. Effect of chromium supplementation on productive and reproductive performances and some metabolic parameters in late gestation and early lactation of dairy cows. Biological Trace Element Research 149(1):42-49.

Khalili, M., A. Foroozandeh, and M. Toghyani. 2011. Lactation performance and serum biochemistry of dairy cows fed supplemental chromium in the transition period. African Journal of Biotechnology 10(50):10304-10310.

Shan, Q., F. Ma, Y. Jin, D. Gao, H. Li, and P. Sun. 2020. Chromium yeast alleviates heat stress by improving antioxidant and immune function in Holstein mid-lactation dairy cows. Anim. Feed Sci. Technol. 269:114635.

Smith, K., M. Waldron, J. Drackley, M. Socha, and T. Overton. 2005. Performance of dairy cows as affected by prepartum dietary carbohydrate source and supplementation with chromium throughout the transition period. Journal of Dairy Science 88(1):255-263.

Yang, W., D. Mowat, A. Subiyatno, and R. Liptrap. 1996. Effects of chromium supplementation on early lactation performance of Holstein cows. Canadian Journal of Animal Science 76(2):221-230.
